# PcrA Dissociates RecA Filaments and the SsbA and RecO Mediators Counterbalance Such Activity

**DOI:** 10.3389/fmolb.2022.836211

**Published:** 2022-02-09

**Authors:** Begoña Carrasco, María Moreno-del Álamo, Rubén Torres, Juan Carlos Alonso

**Affiliations:** Department of Microbial Biotechnology, Centro Nacional de Biotecnología, CNB-CSIC, Madrid, Spain

**Keywords:** ATPase, strand exchange, UvrD, RecA, SsbA, RecO

## Abstract

PcrA depletion is lethal in wild-type *Bacillus subtilis* cells. The PcrA DNA helicase contributes to unwinding RNA from the template strand, backtracking the RNA polymerase, rescuing replication-transcription conflicts, and disassembling RecA from single-stranded DNA (ssDNA) by poorly understood mechanisms. We show that, in the presence of RecA, *circa* one PcrA/plasmid-size circular ssDNA (cssDNA) molecule hydrolyzes ATP at a rate similar to that on the isolated cssDNA. PcrA K37A, which poorly hydrolyses ATP, fails to displace RecA from cssDNA. SsbA inhibits and blocks the ATPase activities of PcrA and RecA, respectively. RecO partially antagonizes and counteracts the negative effect of SsbA on PcrA- and RecA-mediated ATP hydrolysis, respectively. Conversely, multiple PcrA molecules are required to inhibit RecA·ATP-mediated DNA strand exchange (DSE). RecO and SsbA poorly antagonize the PcrA inhibitory effect on RecA·ATP-mediated DSE. We propose that two separable PcrA functions exist: an iterative translocating PcrA monomer strips RecA from cssDNA to prevent unnecessary recombination with the mediators SsbA and RecO balancing such activity; and a PcrA cluster that disrupts DNA transactions, as RecA-mediated DSE.

## Introduction

Superfamily 1A (SF1A) DNA helicases/translocases, which move in the 3′→5′ direction, are ubiquitous (Singleton et al., 2007; Lohman et al., 2008). These enzymes, like those of the Proteobacteria [Rep (only present in γ-Proteobacteria) and UvrD], Actinobacteria (UvrD1 and UvrD2), Firmicutes (PcrA), and Ascomycota (Srs2 and Fbh1) phyla are crucial for DNA repair and repair-by-recombination (Singleton et al., 2007; Lohman et al., 2008; Epshtein, 2015). Genetic data revealed that *Escherichia coli* cells lacking both Rep and UvrD cannot form colonies in rich medium (Taucher-Scholtz et al., 1983), and *Saccharomyces cerevisiae* lacking Srs2 exhibits synthetic lethality with mutations in certain genes [*e.g.*, absence of Sgs1 (counterpart of bacterial RecQ)] (Gangloff et al., 2000), whereas *Mycobacterium* UvrD2 and *Bacilli* PcrA are essential for cell viability (Petit et al., 1998; Williams et al., 2011). In the *Bacilli* Class, a PcrA mutant unable to translocate along DNA (PcrA K37A) is not able to compensate for the lack of the wild-type (*wt*) protein (Petit et al., 1998; Petit and Ehrlich, 2002; Moreno-Del Alamo et al., 2020). Similarly, the UvrD/PcrA-type ATPase domain of *Mycobacterium* UvrD2, but not the *wt* signatures characteristic of the RecQ DNA helicase clade, is essential for cell viability (Sinha et al., 2008; Williams et al., 2011), suggesting that the primary activity, at least of PcrA and UvrD2, is translocating along ssDNA. However, comparative genetics provide little information on the primary cause of PcrA lethality [counterpart of *E. coli* UvrD (UvrD_*Eco*_)] (Petit et al., 1998). PcrA depletion inviability requires RecA and its positive mediators (RecO, RecR) and modulator (RecF), among other RecA accessory functions, but not RecQ or AddAB (counterpart of RecBCD_*Eco*_) (Petit and Ehrlich, 2002; Moreno-Del Alamo et al., 2020). Similarly, in *S. cerevisiae*, the Δ*sgs1* Δ*srs2* synthetic lethality is suppressed by *rad51* inactivation (Krejci et al., 2003; Veaute et al., 2003). By contrast, *E. coli* Δ*uvrD* Δ*rep* rich medium synthetic lethality is suppressed by the rpoB*35 mutation, which alleviates the deleterious consequences of RNA polymerase (RNAP) backtracking and replication-transcription conflict (RTC) formation, but neither Δ*recA* nor ΔrecA rpoB*35 mutations suppress the rich medium synthetic lethality of Δ*uvrD* Δ*rep* cells (Veaute et al., 2005; Guy et al., 2009; Syeda et al., 2016). Unless stated otherwise, the indicated genes and products are of *Bacillus subtilis* origin.

Biochemical analyses of the UvrD_*Eco*_ and PcrA molecular motors revealed that these enzymes have at least five different activities (Singleton et al., 2007; Lohman et al., 2008; Epshtein, 2015). First, PcrA (UvrD_*Eco*_), which physically interacts with UvrB, PolA, and LigA (Sanders et al., 2017), acts at the late stage of the global genome nucleotide excision repair (NER) by unwinding the single-stranded (ss) DNA fragment bearing a distorting lesion, like those induced by UV or its mimetic 4-nitroquinoline 1-oxide (4NQO), with UvrB as a likely candidate to load PcrA onto specific ssDNA sites (reviewed in 3, 17). Unlike UvrD_*Eco*_ (Epshtein, 2015), PcrA does not seem to contribute to mismatch repair. MutS or MutL neither interacts with (Costes et al., 2010) nor stimulates the ATPase activity of PcrA (M.M.del-A. unpublished results). Second, PcrA (UvrD_*Eco*_) and Mfd, which interact with the RNAP, backtracks and pushes forward a stalled RNAP, respectively, to ensure repair of the template strand *via* transcription-coupled repair (TCR) (Deaconescu et al., 2006; Delumeau et al., 2011; Epshtein et al., 2014). Nevertheless, evidence for strand-specific repair mediated by UvrD_*Eco*_/PcrA occurring independently of Mfd-mediated TRC is missing (Adebali et al., 2017; Lindsey-Boltz and Sancar, 2021). Third, PcrA and RnhC, which also physically interact with the RNAP, contribute to alleviating RTCs by removing the RNAP damage sensor of the RNA-DNA hybrids and degrading the RNA, respectively (Delumeau et al., 2011; Lang et al., 2017; Moreno-del Álamo et al., 2021). Fourth, PcrA performs important roles in nudging homologous recombination intermediates toward non-crossover products, and PcrA exhaustion increases the proportion of unsegregated chromosomes by ∼50-fold (Moreno-Del Alamo et al., 2020). Finally, the energy consumed by PcrA (UvrD_*Eco*_) while translocating on ssDNA is used in the removal of proteins residing on the same strand, as the recombinase RecA_*Eco*_, to prevent it from provoking unscheduled recombination during replication fork repair (Veaute et al., 2005; Anand et al., 2007; Park et al., 2010; Fagerburg et al., 2012; Petrova et al., 2015), suggesting that RecA_*Eco*_ may target stalled forks and initiate DNA strand exchange (DSE) when there is no need. However, how PcrA displaces its cognate recombinase and its species-specific coordination and regulation is poorly understood. To understand the pro- and anti-recombination roles of these enzymes, we have characterized *B. subtilis* PcrA.

Among the SF1A DNA helicases/translocases, which promote genome stability by dismantling toxic recombination intermediates, *S. cerevisiae* Srs2 (Srs2_*Sce*_) is the best characterized. The available biochemical information revealed that Srs2_*Sce*_ is recruited to RPA_*Sce*_ clusters embedded between Rad51_*Sce*_ filaments, rather than by interacting with Rad51_*Sce*_ (Burgess et al., 2009; Kaniecki et al., 2017). Then, a multimeric Srs2_*Sce*_ array stimulates the Rad51_*Sce*_ ATPase activity to facilitate its disassembly, but Srs2_*Sce*_ is unable to displace a heterologous recombinase (*e.g.*, RecA_*Eco*_) from ssDNA (Antony et al., 2009; Qiu et al., 2013; Kaniecki et al., 2017). It is poorly understood how enzymes of the γ-Proteobacteria (UvrD_*Eco*_) and Bacilli [*Staphylococcus aureus* (PcrA_*Sau*_), *Geobacillus stearothermophilus* (PcrA_*Gst*_), or *B. subtilis* PcrA] classes are recruited on the ssDNA and how they may disrupt the recombinase nucleoprotein filaments. Various mechanisms have been proposed, but the peculiarities among these distantly related bacteria complicate the understanding of the molecular mechanism of RecA displacement. First, the ATPase activity of UvrD_*Eco*_ and Srs2_*Sce*_ is required to displace RecA_*Eco*_ and Rad51_*Sce*_, respectively, from ssDNA (Krejci et al., 2003; Veaute et al., 2003; Petrova et al., 2015), whereas other authors proposed that the ATPase activity of PcrA_*Sau*_ is not required for RecA_*Eco*_ filament displacement from a small 21-nucleotide (nt) linear poly(dT) ssDNA (dT_21_) (Anand et al., 2007). Second, ATP hydrolysis by RecA_*Eco*_ is crucial for the PcrA_*Gst*_-mediated displacement of the recombinase from 40 nt linear poly(dT) ssDNA (dT_40_) (Fagerburg et al., 2012). In contrast, the RecA_*Eco*_ ATPase activity is not necessary for UvrD_*Eco*_-mediated RecA_*Eco*_ filament displacement from plasmid-size circular ssDNA (cssDNA) (Petrova et al., 2015). Third, PcrA_*Gst*_ may disassemble RecA_*Eco*_ from a dT_40_ *via* a passive (Fagerburg et al., 2012) or repetitive motion on linear dT_40_ ssDNA with PcrA_*Gst*_ actively preventing RecA_*Eco*_ nucleoprotein filament formation (Park et al., 2010). Finally, PcrA_*Sau*_ or PcrA_*Gst*_ can disassemble heterologous RecA_*Eco*_ nucleoprotein filaments formed on short linear dT_21_ or dT_40_ ssDNA (Anand et al., 2007; Park et al., 2010; Fagerburg et al., 2012), but it is unknown whether PcrA can displace its cognate RecA from a plasmid-size cssDNA substrate.

RecA_*Eco*_ and UvrD_*Eco*_ are the bacterial paradigm of recombinases and SF1A DNA translocases/helicases, and these enzymes have provided the mechanistic models in the field. However, bacteria of the *Bacilli* class have evolutionarily diverged from those of the γ-Proteobacteria Class (represented by *E. coli*) more than 2 billion years ago, and such a divergency is also encompassed by differences in the mode of action of the recombinase. First, *Bacilli* RecA, in the ATP bound form (RecA·ATP), cannot catalyze plasmid-size DSE in the absence of mediators, but RecA·dATP or RecA_*Eco*_·ATP does (Lovett and Roberts, 1985; Steffen et al., 2002; Cox, 2007; Kowalczykowski, 2015). Second, *B. subtilis* RecA·ATP neither nucleates nor polymerizes on the SsbA-cssDNA complexes (Carrasco et al., 2008; Manfredi et al., 2008), but RecA·dATP or RecA_*Eco*_·ATP polymerizes in the presence of SsbA or SSB_*Eco*_, respectively (Cox, 2007; Yadav et al., 2012; Kowalczykowski, 2015). Third, a two-component mediator (SsbA and RecO) is necessary to activate RecA·ATP to catalyze plasmid-size DSE *in vitro* (or SsbA, RecO, and RecR *in vivo*), whereas DSE is further stimulated by RecA·dATP or RecA_*Eco*_·ATP in the presence of mediators (Cox, 2007; Manfredi et al., 2008; Yadav et al., 2012; Carrasco et al., 2015; Kowalczykowski, 2015). Fourth, the negative modulators RecX and RecU, which physically interact with RecA (Carrasco et al., 2005; Cárdenas et al., 2012), promote RecA disassembly (Le et al., 2017; Serrano et al., 2018), and it is predicted that PcrA, which co-purifies with RecA in tandem affinity purification (Tap-tag) (Costes et al., 2010), may promote RecA disassembly from ssDNA (Moreno-del Álamo et al., 2021). Finally, a *Bacilli* PcrA monomer translocates along ssDNA in a processive manner, but multiple monomers are required to unwind duplex DNA (Singleton et al., 2007; Lohman et al., 2008). It is unknown whether PcrA displaces its cognate RecA from a cssDNA substrate, if PcrA loads on cssDNA at a SsbA region, if PcrA affects the ATP hydrolysis rate of a cognate RecA in the presence of the SsbA and RecO mediators, and how the activities of these proteins are coordinated.

To understand the pro- and anti-recombination roles of PcrA, the molecular mechanism of RecA nucleoprotein filament disassembly by PcrA, and how RecA mediators (SsbA and RecO) may modulate such activity, we have performed genetic and biochemical assays. We have shown that PcrA depletion lethality and the sensitivity to 4NQO are suppressed by *recA* or *recO* inactivation, but only 4NQO sensitivity is suppressed by *mfd* inactivation. Thus, we asked whether PcrA, as an anti-recombinase, specifically displaces a cognate RecA from ssDNA and inhibits DSE, and if the two-component mediator (SsbA and RecO) contributes to balance such activity. We show that *circa* one PcrA/cssDNA molecule catalytically removes RecA *via* an active mechanism that requires PcrA-mediated ATP hydrolysis. SsbA inhibits the ATPase activity of PcrA, but blocks RecA-mediated ATP hydrolysis. RecO antagonizes SsbA to stimulate the ATPase activity of RecA, and only partially stimulates the ATPase of PcrA. A PcrA cluster, in the presence of the two-component mediator (SsbA and RecO), displaces RecA·ATP from ssDNA and inhibits DSE, but RecA·dATP, which shows an increased filament stability, is only partially displaced by PcrA from ssDNA. The two-component mediator differentially balances both PcrA activities.

## Materials and Methods

### Bacterial Strains and Plasmids

All *B. subtilis* strains were isogenic derivatives of BG214 (*wt* strain), as listed in Supplementary Table S1. The *pcrA*-*ssrA* and *sspB* cassettes of the degron *pcrA*_T_ strain were moved into the Δ*mfd* or *recO*16 context, or the Δ*recA* mutation on the *pcrA*-*ssrA sspB* background by SPP1-mediated generalized transduction to reconstruct the strains. The *pcrA*-*ssrA* and *sspB* cassettes and the Δ*mfd* mutation were also moved in a successive step by natural chromosomal transformation to reconstruct the Δ*mfd pcrA*_T_ background, as described by Moreno-Del Alamo et al. (2020).

*E. coli* BL21(DE3) (pLysS) cells bearing the pCB722 (*ssbA*)*,* pCB669 (*recO*), pCB1020 (*radA*), pCB1035 (*radA* C13A), or pCB906 (*rarA*) plasmid, or *E. coli* M15 (pREP4) cells bearing the pCB1229 (*pcrA*), or pCB1230 (*pcrA* K37A, Walker A mutant variant) plasmid were used to overproduce the SsbA, RecO, RadA/Sms, RadA/Sms C13A, RarA, PcrA, or PcrA K37A proteins, respectively (Carrasco et al., 2008; Manfredi et al., 2008; Carrasco et al., 2018; Torres et al., 2019; Moreno-del Álamo et al., 2021). *B. subtilis* BG214 cells bearing the pBT61 (*recA*) plasmid were used to overproduce RecA (Gassel and Alonso, 1989).

### Survival Assays

Exponentially growing *pcrA*-*ssrA sspB* (*pcrA*_T_) cells were plated in rich medium onto agar plates containing isopropyl-β-D thiogalactopyranoside (IPTG) to induce SspB expression from a LacI regulated promoter (Griffith and Grossman, 2008; Merrikh et al., 2015). SspB then bound the SsrA peptide tag and rapidly delivered the tagged PcrA-SsrA protein to the *B. subtilis* ClpXP protease for degradation [PcrA degron (*pcrA*_T_) strain] (Keiler et al., 1996; Griffith and Grossman, 2008). PcrA degron cultures (*pcrA*_T_ Δ*mfd*, *pcrA*_T_ *recO*16, or *pcrA*_T_ Δ*recA*) were grown to OD_560_ = 0.4. The cultures were serially diluted and appropriate dilutions plated on LB agar plates alone or with 500 μM IPTG (Calbiochem). Plates were incubated overnight (16–18 h, 37°C), and the percentage of colony-forming units (CFUs) in LB agar plates containing IPTG was measured. The mean ± standard error of the mean (SEM) was calculated using the R software (The R Foundation), and a Student’s *t-*test was performed to denote the threshold of significance.

**FIGURE 1:**
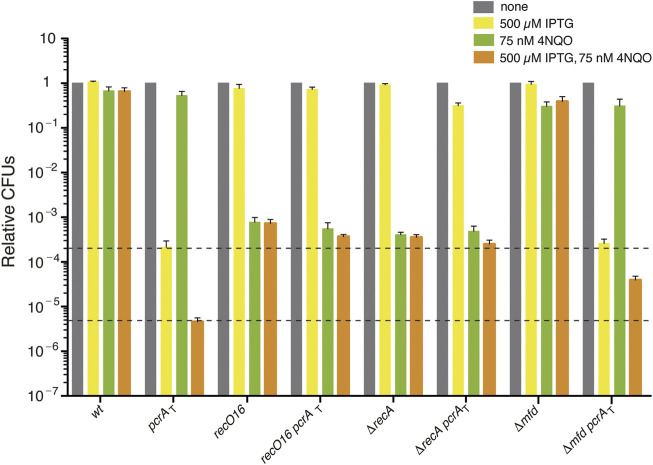
The sensitivity of PcrA depleted cells to 4NQO is partially suppressed by *recO*, *recA*, or *mfd* inactivation. Log phase cultures of *wt*, single (*pcrA*_T_, *recO*16, Δ*recA*, or Δ*mfd*) and double mutant (*pcrA*_T_ *recO*16, *pcrA*_T_ Δ*recA*, or *pcrA*_T_ Δ*mfd*) strains were diluted, plated on LB agar (grey bars), LB agar + 500 μM IPTG (yellow bars), LB agar + 75 nM 4NQO (green bars), or LB agar + 500 μM IPTG + 75 nM 4NQO (orange bars) and incubated overnight at 37°C. Experiments were performed at least four times. The dotted lines mark the upper and lower limit of the cell viability rate upon PcrA depletion. Data are shown as mean fractional survival ±SEM.

The UV mimetic 4NQO was from Sigma-Aldrich. Cell sensitivity to chronic 4NQO exposure was determined by growing cultures to OD_560_ = 0.4 and plating appropriate dilutions on LB agar plates containing 4NQO (75 nM) or IPTG (500 μM) and 4NQO (75 nM) as described (Moreno-del Álamo et al., 2021). Plates were incubated overnight (16–18 h, 37°C), and the number of CFUs was determined (Figure 1). Experiments were conducted independently at least four times. Fractional survival data are shown as mean ± SEM. Statistical analysis was performed with a two-tailed Student’s t-test. For experiments involving more than two groups, a one-way analysis of variance (ANOVA) was performed. For all tests, a *p* value of < 0.1 was considered significant. All statistical analyses were performed using the R software (The R Foundation).

### Enzymes, Reagents, DNA, Protein, and DNA and Protein Purification

All chemicals used were of analytical grade. DNA polymerases, DNA restriction enzymes, and DNA ligase were from New England Biolabs, and polyethyleneimine, DTT, ATP, dATP, and ATPγS were from Sigma-Aldrich (Merck).

pGEM3 Zf(+) (Promega Biotech Ibérica) was used to construct the substrate for *in vitro* DSE assays. The sequence of the oligonucleotides used to construct the DSE substrates is indicated in the 5′→3′ polarity: *lds*_*hom*_, CAT​GTT​CAG​CGG​CAG​CGG​ATA​GCG​GGA​AAG​CGG​ATA​GCG​GCAA​GCG​GAA​AGC​GGA​TAG​CGG​TAA​GCG​GAA​GCG​GTT​A; *lds*_*het*_, CATGTTTGGCGAAGGCGA​ATG​GCG​ATA​GGC​GAA​AGG​CGA​ACG​GCG​ATA​GGC​GAA​GGG​CGA​TAG​GCG​ACG​GCGACTAC (variant with 42 mismatches [54.4% sequence divergence]). These oligonucleotides and their complements were joined to *Afl*II-cleaved 3199-base pairs (bp) pGEM3 Zf(+) to render 3276 bp pGEM3_hom_ (*lds*_*hom*_) or pGEM3_*het*_ (*lds*_*het*_) as described by Carrasco et al. (2019). The 3353 bp pGEM3_het-ins_ plasmid, which derives from pGEM3_*het*_, contained the 77 bp heterologous sequence also at the *Eco*RI-cleaved site as described by Carrasco et al. (2015). The 4374 bp pGEM-1.2 dsDNA contains a 1175 bp heterologous DNA segment at the *Afl*II-cleaved site of pGEM3Zf (+) DNA. Thus, linearization with *Eco*RI places the heterology at the 3′-end, with *Pst*I at the 5′-end on the (−) strand of the duplex DNA. The 3199 bp pGEM3Zf (+), 4375 bp pGEM-1.2, 3276 bp pGEM _het_, and 3353 bp pGEM3_het-ins_ and its ssDNA variants were purified as described by Carrasco et al. (2005).

The 3′-tailed Holliday Junction (HJ) DNA structure (HJ-lead) was assembled by annealing J3-1, J3-2-110, J3-3, and J3-4, whose sequences are indicated in the 5′ → 3′polarity: J3-1, CGC​AAG​CGA​CAG​GAA​CCT​CGA​GAA​GCT​TCC​GGT​AGC​AGC​CTG​AGC​GGT​GGT​TGA​ATTCCT​CGA​GGT​TCC​TGT​CGC​TTG​CG; J3-2-110, CGC​AAG​CGA​CAG​GAA​CCT​CGA​GGA​ATT​CAAC​CAC​CGC​TCA​ACT​CAA​CTG​CAG​TCT​AGA​CTC​GAG​GTT​CCT​GTC​GCT​TGC​GAA​GTC​TTTC​CGG​CAT​CGA​TCG​TAG​CTA​TTT; J3-3, CGC​AAG​CGA​CAG​GAA​CCT​CGA​GTC​TAG​ACTGCA​GTT​GAG​TCC​TTG​CTA​GGA​CGG​ATC​CCT​CGA​GGT​TCC​TGT​CGC​TTG​CG; J3-4, CGCAAGC​GAC​AGG​AAC​CTC​GAG​GGA​TCC​GTC​CTA​GCA​AGG​GGC​TGC​TAC​CGG​AAG​CTT​CTCGAG​GTT​CCT​GTC​GCT​TGC​G. DNA concentrations were established using the molar extinction coefficients of 8780 and 6500 M^−1^ cm^−1^ at 260 nm for ssDNA and dsDNA, respectively, and are expressed as moles of nucleotides.

SsbA (18.7 kDa), RecO (29.3 kDa), PcrA (83.5 kDa), PcrA K37A (83.4 kDa), RecA (38.0 kDa), RadA/Sms (49.4 kDa), RadA/Sms C13A (49.4 kDa), and RarA (46.3 kDa) proteins were expressed and purified as described (Carrasco et al., 2005; Carrasco et al., 2008; Manfredi et al., 2008; Carrasco et al., 2018; Torres et al., 2019; Moreno-del Álamo et al., 2021). All proteins were purified to more than 98% homogeneity. Purified SsbA, RecO, PcrA, PcrA K37A, RecA, RadA/Sms, RadA/Sms C13A, or RarA in the presence of 5 mM ATP and 10 mM Mg(CH_3_COO)_2_ lack any protease, exo- or endonuclease activity in pGEM3 Zf(+) ssDNA, or dsDNA. The corresponding molar extinction coefficients for SsbA, RecO, RecA, RadA/Sms, PcrA, and RarA were calculated at 280 nm as 11400; 19600; 15200, 24930; 70375; and 29465 M^−1^ cm^−1^, respectively (Carrasco et al., 2005). Protein concentrations were determined using the above molar extinction coefficients. RecA, RecO, PcrA, and PcrA K37A are expressed as moles of monomeric, SsbA as tetrameric, and RadA/Sms and RadA/Sms C13A as hexameric proteins. The experiments were performed under optimal RecA conditions in buffer A [50 mM Tris-HCl (pH 7.5), 1 mM DTT, 50 mM NaCl, 10 mM Mg(CH_3_COO)_2_, 50 μg/ml bovine serum albumin (BSA), and 5% glycerol]. When RecA was omitted, the experiments were performed in buffer B [50 mM Tris-HCl (pH 7.5), 1 mM DTT, 80 mM NaCl, 10 mM Mg(CH_3_COO)_2_, 50 μg/ml BSA, and 5% glycerol]. The site size of RecA is 1 monomer/3 nt, PcrA requires 8 nt for loading on ssDNA, and SsbA has two binding modes (binds ssDNA in the fully wrapped (SSB_65_) or in the (SSB_35_) mode] (Chen et al., 2008; Shereda et al., 2008; Manfredi et al., 2010; Moreno-del Álamo et al., 2021).

### Protein–Protein Interactions

*In vitro* protein–protein interactions were analyzed by immuno-dot blot assays (Walsh et al., 2012), using the Bio-Dot apparatus (Bio-Rad). Briefly, increasing amounts of PcrA, BSA (as a negative control), and RarA (as a positive control of interaction) (500–2000 ng) were applied to a pre-wetted nitrocellulose membrane in 1X phosphate-buffered saline (PBS, 137 mM NaCl; 2.7 mM KCl; 10 mM Na_2_HPO_4_; 1.8 mM KH_2_PO_4_, pH 7.4). After blocking with PBS containing 5% (w/v) skimmed milk powder, the membrane was incubated for 6 h with 400 ng RecA in binding solution [PBS, 0.5% (w/v) skimmed milk powder and 0.1% (v/v) triton X-100] at 4°C. The membrane was then incubated overnight at 4°C with anti-RecA polyclonal antibody (dilution 1:5000) and subsequently with the secondary antibody anti-rabbit IgG conjugated with peroxidase (dilution 1:5000) for 1 h at room temperature. The interactions were visualized by staining the membrane with Clarity™ Western ECL Substrate kit (Bio-Rad). The images were obtained and processed with the ChemiDoc Imaging System and the Image Lab software (Bio-Rad).

His-tagged PcrA was used to test the strength of a PcrA-RecA interaction. His-tagged PcrA (1 μg), RecA (1 μg), or both, were incubated with 50 μL of the Ni^2+^ matrix in buffer A containing 5 mM ATP, and the flow-through (FT) was collected. The Ni^2+^ matrix was washed four times with 500 μL of buffer A containing 20 mM imidazole [the first (W1) and the last (W4)] were collected) and eluted (E) with buffer A containing 1 M NaCl and 0.4 M imidazole. The collected protein fractions were separated by 12.5% (w/v) SDS-PAGE.

### Nucleotide Hydrolysis Assays

The cssDNA-dependent ATP hydrolysis activity of PcrA, its variant (PcrAK37), and RecA was assayed *via* a NAD/NADH coupled spectrophotometric enzyme assay (Yadav et al., 2012). The rate of cssDNA-dependent PcrA-mediated ATP hydrolysis and the time needed to reach a steady-state ATP hydrolysis (lag time) were measured in buffer A containing 5 mM ATP, but RadA/Sms C13A-mediated ATP hydrolysis was measured in buffer B (Yadav et al., 2012). The reactions additionally contained the NADH enzyme mix (300 μM NADH, 100 U/ml of lactate dehydrogenase, 500 U/ml pyruvate kinase, and 2.5 mM phosphoenolpyruvate) and had a volume of 50 μL (30 min, 37°C). The order of addition of 3199 nt pGEM3 Zf(+) cssDNA and purified proteins is indicated. When the nucleotide hydrolysis assay was initiated with the first proteins and at the indicated time, a second protein is added, a slight decline in absorption, caused by a dilution effect, was observed. Thus, the data following the addition of a second protein have been corrected. The ATPase activity was determined by monitoring the disappearance of absorbance at 340 nm due to NADH conversion to NAD, using a Shimadzu CPS-20A dual-beam spectrophotometer. A standard curve with known amounts of NADH was obtained and used to convert the drop-in absorbance/time to ADP concentration/time (Yadav et al., 2012). Data obtained from ATP hydrolysis were converted to ADP and plotted as a function of time (Yadav et al., 2012). The lag time, which represents the delay in reaction progress relative to a theoretical reaction curve that lacks a lag time, was derived from the time intercept of a linear regression line fit to the steady-state portion of data in ATP hydrolysis assays (Yadav et al., 2012).

### RecA-Mediated DNA Strand Exchange

The linear dsDNA substrate (*lds*) (*Kpn*I-linearized pGEM3 Zf(+), pGEM3_*hom*_, or pGEM3_*het*_ (*lds*_*het*_), or *Ps*tI-linearized or *EcoR*I-linearized pGEM-1.2) and a homologous 3199 nt long cssDNA (10 μM in nt) were incubated with the indicated concentrations of protein or protein combinations in buffer A containing 5 mM ATP, dATP, or ATPγS for 60 min at 37°C in a final volume of 20 μL. A (d)ATP regeneration system (8 units/ml creatine phosphokinase and 8 mM phosphocreatine) was included in all recombination reactions. After incubation, samples were deproteinized and fractionated by 0.8% agarose gel electrophoresis with ethidium bromide (Ayora et al., 2002). The signal of the remaining *lds* and the appearance of joint molecule (*jm*) intermediates and the final recombination product (a nicked circular [*nc*] or a [*prd*]) were quantified from gels using a Geldoc (BioRad) system and the ImageJ software (NIH) (Manfredi et al., 2008). When indicated, the sum of *jm* and *nc* is shown as % recombination.

## Results and Discussion

### Inactivation of *recA* or *mfd* Increases Survival to 4NQO-Induced DNA Damage Upon PcrA Depletion

Previously, it has been shown that: 1) PcrA/UvrD_*Eco*_ contributes to NER by displacing the excised damaged DNA segment; 2) PcrA/UvrD_*Eco*_ interacts with and induces RNAP backtracking to alleviate RTCs; 3) Mfd_*Eco*_, which interacts with the RNAP, contributes to TCR and pushes the RNAP forward (anti-backtracking activity) to correctly position its active site without interaction with the DNA; 4) inactivation of *mfd*_*Eco*_ suppresses the sensitivity to UV radiation in the Δ*uvrD*_*Eco*_ context; and 5) PcrA co-purifies with RecA as revealed by Tap-tag experiments (see Introduction). The mechanistic basis of the interplay between PcrA and Mfd or RecA remains poorly understood.

To understand the primary contribution of PcrA to NER, TCR, and RTCs or to limit unwanted recombination, the Δ*mfd pcrA*_T_ and Δ*recA pcrA*_T_ strains were exposed to the UV mimetic 4NQO and the survival rate analyzed under selective depletion of PcrA, with the *recO*16 *pcrA*_T_ strain taken as a control in these experiments. We have previously reported that, upon PcrA depletion (by IPTG), in the Δ*mfd pcrA*_T_ (BG1875 strain), the lethality is partially suppressed, but the resulting colonies are minute and with an area ∼19-fold smaller than in the presence of PcrA (Moreno-Del Alamo et al., 2020), although our preliminary analyses appear now to contradict this previous report. To confirm these results, we reconstructed the Δ*mfd pcrA*_T_, with the *recO*16 *pcrA*_T_ and Δ*recA pcrA*_T_ strains as controls, and the phenotype of the newly constructed strains was compared with the previously reported strains (see Supplementary Table S1) (Moreno-Del Alamo et al., 2020). As revealed in Supplementary Materials Annex 1, in the presence of IPTG (500 μM), PcrA depletion lethality was suppressed by >1400-fold in the new and old *recO*16 *pcrA*_T_ or *ΔrecA pcrA*_T_ strains when compared with the *pcrA*_T_ strain. Thus, the data from the former BG1715 (*recO*16 *pcrA*_T_) and BG1877 (*ΔrecA pcrA*_T_) strains are plotted in Figure 1 (yellow bars). When the newly constructed Δ*mfd pcrA*_T_ (BG1923) and the previous BG1875 strain were analyzed, a different outcome was observed. As described in Supplementary Materials Annex 1, PcrA depletion inviability did not require the dsDNA translocase Mfd when the new BG1923 strain was tested. The presence of the Δ*mdf* mutation and the *wt pcrA*_T_ cassettes was confirmed by sequencing and corroborated by the construction of a new set of strains. It is known that Mfd may function as an anti-mutator in DNA damage-induced mutagenesis, and it appears to function as a mutator for spontaneous mutagenesis (Witkin, 1994; Lindsey-Boltz and Sancar, 2021). Then, it is likely that unselected mutations may account for the minute colony formed with the former Δ*mfd pcrA*_T_ BG1875 strain but not with the BG1923 strain. Here, any further analysis of the Δ*mfd pcrA*_T_ cells was performed with the newly constructed BG1923 strain (Supplementary Materials Annex 1).

To understand the primary cause of PcrA lethality in the presence of replicative stress (a limiting concentration of the UV mimetic 4NQO), the strains listed in Table S1 were grown in rich LB medium to OD_560_ = 0.4 and plated on plates containing 500 μM IPTG, 75 nM 4NQO, or both. The 4NQO-induced bulky lesions on the template strand are specifically removed from duplex DNA by global-genome NER and by the minor TCR sub-pathways (Selby and Sancar, 1994; Truglio et al., 2006). If 4NQO-induced lesions escape these specialized repair sub-pathways (*e.g.*, they are in ssDNA regions), homologous recombination functions should contribute to remodeling the stalled fork and circumvent or bypass the lesion or repairing the double-strand breaks (Kowalczykowski, 2015).

The presence of 75 nM 4NQO barely compromised the viability of *wt* cells (by ∼1.4-fold) (Figure 1, grey *vs.* green bars). Cell survival was not affected in the *pcrA*_T_ strain (by ∼1.2-fold) and slightly reduced in the Δ*mfd* strain (by ∼2.5-fold [*p* < 0.05]). When *pcrA*_T_ cells were plated on plates containing 500 μM IPTG and 75 nM 4NQO, the survival was reduced by ∼46-fold when compared to the only IPTG condition (Figure 1) (Moreno-del Álamo et al., 2021). The survival of *pcrA*_T_ Δ*mfd* cells plated on plates containing IPTG and 4NQO was significantly increased (by ∼8-fold) when compared to the *pcrA*_T_ control strain (Figure 1, orange bars). Similar results were observed when other newly constructed *pcrA*_T_ Δ*mfd* clones were tested (Supplementary Materials Annex 1). It is likely that: 1) PcrA depletion in otherwise *wt* cells, which renders a complex phenotype and overlapping defects (unwanted toxic recombination intermediates, NER impairment, and inability to remove RTCs), is responsible for the poor survival upon 4NQO exposure; and 2) the Δ*mfd* defect, *via* eliminating control over PcrA-mediated RNAP backtracking, should relieve the 4NQO-sensitivity of *pcrA*_T_ Δ*mfd* cells upon IPTG addition.

Inactivation of *recO* or *recA* strongly impaired, by ∼1300- and ∼2500-fold [*p* < 0.01] the survival of single mutant strains, when compared to the *wt* control in the presence of 75 nM 4NQO (Figure 1, grey *vs.* green bars). The survival of *pcrA*_T_ Δ*recA* or *pcrA*_T_ *recO*16 cells plated on plates containing IPTG and 4NQO was slightly reduced (by ∼1.9-fold or ∼1.4-fold, respectively) when compared to the parental Δ*recA* or *recO*16 control strain (Figure 1, orange bars). However, it was significantly increased (by ∼80- and ∼55-fold, respectively) when compared to the *pcrA*_T_ parental strain (Figure 1, orange bars). It is likely that PcrA, whose inviability requires RecO and RecA, may prevent RecO and RecA from provoking unnecessary recombination during 4NQO-induced replication stress. Understanding the role of Mfd in concert with PcrA in the TCR sub-pathways would require further studies before analyzing their interplay with RecA and RecO. To know whether PcrA disassembles a cognate RecA nucleoprotein filament in the presence or absence of mediators (RecO and SsbA), biochemical assays were undertaken.

### PcrA Hydrolyzes ATP at a Similar Rate in the Presence or Absence of RecA

Previously, it has been shown that: 1) PcrA_*Gst*_ collides with and caps heterologous RecA_*Eco*_ filament growth on a 40 nt long linear poly(dT) ssDNA (dT_40_), leading to passive RecA_*Eco*_ disassembly (Fagerburg et al., 2012); and 2) PcrA_*Gst*_ bound to a duplex junction actively dismantles a preformed RecA_*Eco*_ nucleoprotein filament from the 3′-distal end of the dT_40_ DNA by an active mechanism (Park et al., 2010). To test whether these discrepancies can be attributed to the use of different methodologies, different substrates, or heterologous proteins, PcrA and its cognate recombinase RecA were purified, and the ATP hydrolysis rate was measured using plasmid-size cssDNA (3199 nt long cssDNA). As RecA cannot disrupt the spontaneously folded duplex junctions and other secondary structures, non-contiguous RecA filaments should be assembled on the cssDNA. The steady-state rate of ATP hydrolysis and the lag time observed for achieving this rate, which provides information on the mechanism used by PcrA to displace RecA, was measured (see *Materials and Methods*).

**FIGURE 2:**
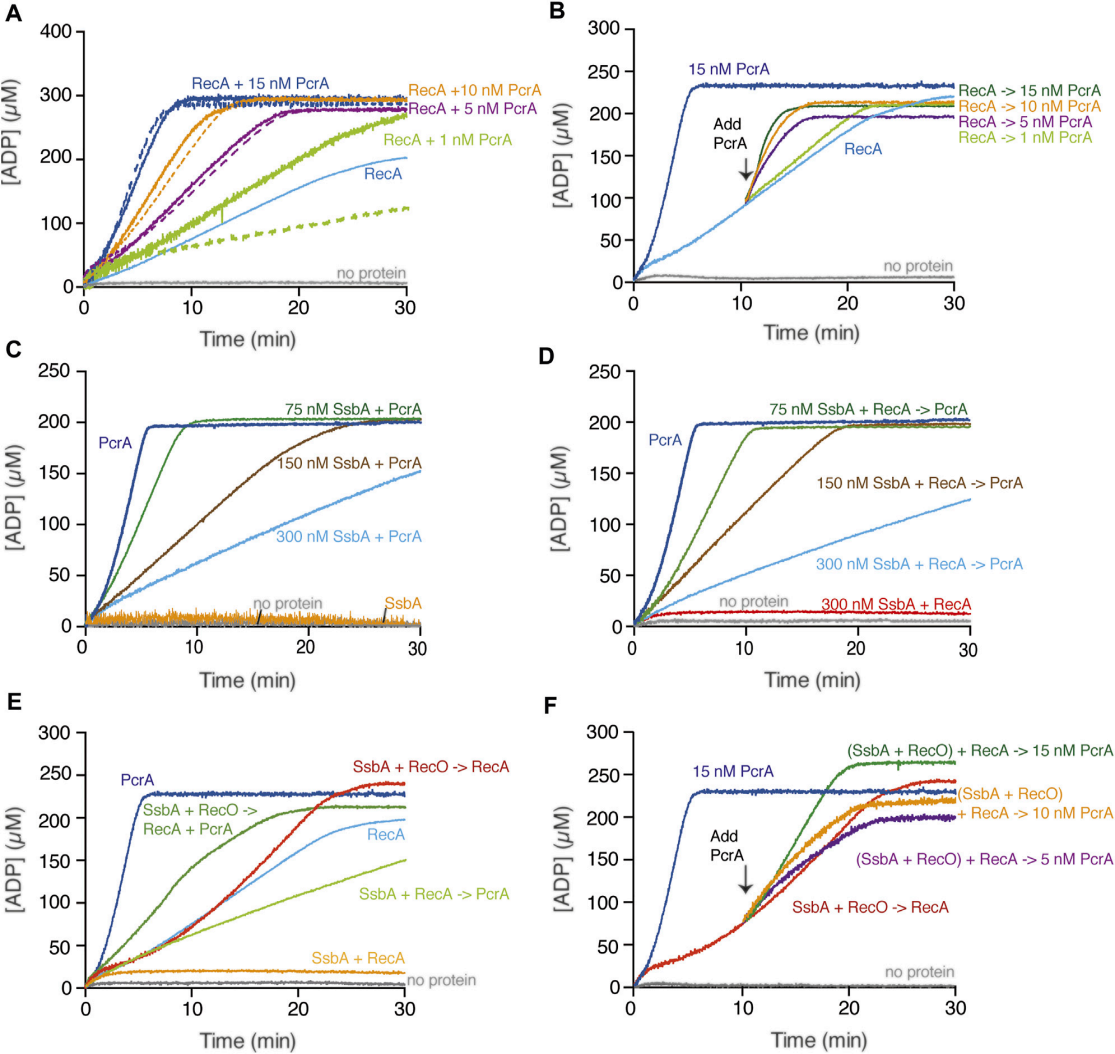
PcrA translocates on the ssDNA in the presence or absence of RecA. **(A,B)** PcrA (1–15 nM), RecA (800 nM), or both proteins were incubated with cssDNA (10 μM in nts) in buffer A containing 5 mM ATP **(A)**; or RecA and cssDNA were pre-incubated (10 min, 37°C) in buffer A containing 5 mM ATP, and then increasing PcrA concentrations were added **(B)**. The grey line denotes the control reaction corresponding to the ATPase assay in the absence of any protein, and the broken lines represent the presence of only PcrA at the indicated concentration **(A,B)**. **(C)** The cssDNA was incubated with fixed PcrA (15 nM) and increasing SsbA concentrations in buffer A containing 5 mM ATP. **(D)** The cssDNA was pre-incubated with fixed RecA and increasing SsbA concentrations (5 min, 37°C) in buffer A, and then PcrA and 5 mM ATP were added. **(E)** The cssDNA was pre-incubated with fixed SsbA (300 nM) and RecO (100 nM) or RecA (5 min, 37°C) in buffer A containing 5 mM ATP and then PcrA (15 nM), RecA (800 nM) or both, and 5 mM ATP were added. **(F)** The cssDNA was pre-incubated with RecA, SsbA, and RecO (10 min, 37°C) in buffer A containing 5 mM ATP, and then PcrA (5–15 nM) was added. Buffer A contained the NADH enzyme mix. The ATPase activity was measured (30 [or 20] min, 37°C). Representative graphs are shown here, and the determined K_cat_ is described in the text.

RecA·ATP cooperatively binds cssDNA to form a dynamic helical nucleoprotein filament by monomer addition to both ends, although it occurs faster at the 3′-end, with a preferential filament growth in the 5′→3′ direction (Cox, 2007; Kowalczykowski, 2015; Bell and Kowalczykowski, 2016). ATP hydrolysis throughout the filament is used to redistribute RecA because it allows the dissociation of RecA·ADP protomers predominantly from filament ends (Cox, 2007; Kowalczykowski, 2015; Bell and Kowalczykowski, 2016). The steady-state rate of ATP hydrolysis by RecA, which is reached without any lag phase, was 9.5 ± 1.9 min^−1^ (Figure 2A, light blue line). A similar K_cat_ for RecA was previously reported (Steffen and Bryant, 1999; Yadav et al., 2014).

PcrA binds ssDNA with an apparent binding constant (K_Dapp_) of ∼1.5 nM (Moreno-del Álamo et al., 2021). In the presence of cssDNA, ATP hydrolysis by 5 nM PcrA (1.6 PcrA monomers/cssDNA) reached a steady-state rate without any lag time and with a K_cat_ of 1430 ± 188 min^−1^ (Figure 2A, broken purple line). Similar results were previously reported (Moreno-del Álamo et al., 2021). ATP hydrolysis is required for efficient and processive PcrA translocation along ssDNA (∼270 nt/s) in the 3′→5′ direction (Niedziela-Majka et al., 2007; Singleton et al., 2007).

Based on the fact that the maximal rate of ATP hydrolysis by PcrA is ∼150-fold higher than that of RecA, we can envision several outcomes when analyzing whether PcrA affects RecA redistribution: 1) a large decrease in ATP consumption if RecA filamented on the cssDNA impedes PcrA translocation on ssDNA; 2) a sum of their individual activities if both can co-exist on the cssDNA; 3) a greater ATP hydrolysis rate than the sum of their individual activities if PcrA stimulates the ATP hydrolysis rate of RecA; or 4) a comparable ATP hydrolysis rate to that of PcrA alone if PcrA translocation displaces RecA bound to ssDNA. To uncover that, ATP hydrolysis assays in the presence of increasing PcrA (1–15 nM) and fixed cssDNA (10 μM in nt or 3.1 nM in molecules) and RecA (800 nM) concentrations were performed (Figure 2A).

In the presence of very limiting PcrA (1 nM, 0.32 PcrA monomers/cssDNA molecule) and a sub-saturating RecA (1 RecA monomer/12.5 nt) concentration relative to cssDNA, the rate of ATP hydrolysis was marginally increased above that seen with RecA alone (Figure 2A, light green and light green broken line *vs*. light blue line). As PcrA concentration increased to 1.6 PcrA/cssDNA molecule (5 nM, 1 PcrA/2000 nt), the slope of the curve of ATP hydrolysis was comparable to that of PcrA alone (Figure 2A, purple and purple broken line *vs*. light blue line). Similar results were observed in the presence of 3.2 or 4.8 PcrA monomers/cssDNA molecule (Figure 2A, orange and dark blue and orange or dark blue broken lines *vs*. light blue line). Please be aware that we assumed that there is no protein free in solution. Thus, our calculated stoichiometries may be overestimated by factor 2.

From these data, it is likely that: 1) less than one PcrA monomer/cssDNA molecule is not sufficient for RecA displacement because PcrA turnover may provide time for RecA re-loading; 2) *circa* one PcrA monomer/cssDNA molecule is necessary to prevent RecA nucleoprotein filament formation on plasmid-size cssDNA perhaps by iterative RecA displacement form the cssDNA; and 3) *circa* 3 or more PcrA monomers/cssDNA molecule cannot further stimulate the ATP hydrolysis rate when compared to one PcrA alone, indicating that there is no significant rebinding of RecA to the cssDNA. In *E. coli*, however, *circa* 50 UvrD monomers/cssDNA molecule are required to operate in a specialized RecA disassembly mode after a lag phase longer than 25 min (Petrova et al., 2015).

### RecA Transiently Interacts With but It May Not Recruit PcrA

How does PcrA bind ssDNA on the RecA-cssDNA complexes? We can envision that: 1) RecA, which co-purifies with PcrA by Tap-tag experiments (Costes et al., 2010), may recruit PcrA on the cssDNA; 2) PcrA, which has a size site of 8–10 nt (Singleton et al., 2007), might bind naked regions between non-contiguous RecA filaments because sub-stoichiometric RecA concentrations (1 RecA/12.5 nt relative to cssDNA) were used in the experiments presented (see Figure 2A); and 3) PcrA might bind secondary structures on cssDNA. To distinguish between these possibilities, we first analyzed whether PcrA physically interacts with RecA using immuno-dot blot assays (Walsh et al., 2014). In a second step, if both proteins interact, we aim to address the ionic strength for such interaction by measuring RecA retention by His-tagged PcrA in a Ni^2+^ matrix at different NaCl concentrations (Torres and Alonso, 2021). As revealed in Supplementary Annex 2, the interaction of PcrA with RecA by immuno-dot blot assays required an excess of the former protein (Supplementary Figure S1A). His-tagged PcrA-bound Ni^2+^ matrix, however, could not retain RecA (Figure S1B) even in the presence of 50 mM NaCl, the NaCl concentration used in the ATPase assays (Figure 2). It is likely that PcrA transiently interacts with RecA.

To test the second hypothesis, we have used stoichiometric RecA concentrations (3000 nM, 1 RecA/3 nt) relative to cssDNA and tested whether the PcrA-RecA interaction displaces the latter, thus stimulating the ATP hydrolysis rate. Alternatively, PcrA bound to the ssDNA at secondary structures simply strips RecA from cssDNA. In the presence of 5 nM PcrA (1.6 PcrAs/cssDNA molecule), the slope at maximal ATP hydrolysis was not informative because it was similar to the one of PcrA or RecA bound to cssDNA (Supplementary Figure S2A, light blue, light blue broken *vs*. orange line), suggesting that PcrA transiently interacts with RecA but is unable to stimulate the ATP hydrolysis activity of RecA. Unlike PcrA, Srs2_*Sce*_ interacts with and stimulates the ATP hydrolysis activity of Rad51_*Sce*_ (Antony et al., 2009; Qiu et al., 2013; Kaniecki et al., 2017).

In the presence of 3.2 (or 4.8) PcrAs/cssDNA molecule, the maximal rate of ATP hydrolysis reached a steady-state rate of ATP hydrolysis without a lag phase, and the slope was comparable to that of PcrA alone (Supplementary Figure S2A, purple, green *vs*. purple and green broken lines). It is likely that: 1) the iterative motion of *circa* 1 to 3 PcrAs on cssDNA is sufficient to prevent reassembly of saturating RecA concentrations and 2) PcrA is not recruited to sites vacated by RecA·ADP because a lag phase was not observed. Since PcrA binds ssDNA with >100-fold higher affinity than RecA and displaces its nucleoprotein filaments in a buffer containing 50 mM NaCl (Singleton et al., 2007; Bell and Kowalczykowski, 2016), we consider it unlikely that a RecA nucleoprotein filament recruits PcrA onto cssDNA at the ionic strength used in our experiments.

### PcrA Displaces Preformed RecA Nucleoprotein Filaments

To further evaluate whether PcrA is recruited upon interaction with RecA and if this promotes a rapid RecA redistribution, the rate of ATP hydrolysis of a preformed RecA-cssDNA complex (1 RecA/12.5 nt) was measured (10 min, 37°C), followed by the addition of increasing PcrA concentrations and measurement for 20 min longer. In the presence of very limiting PcrA (0.32 PcrAs/cssDNA molecule), the steady-state rate of ATP hydrolysis showed a slope similar to RecA alone (Figure 2B, light green *vs*. light blue). As PcrA concentration increased, the maximal rate of ATP hydrolysis significantly increased without any obvious lag phase (Figure 2B). In the presence of 1.6 PcrAs/cssDNA molecule, the steady-state rate of ATP hydrolysis showed a slope similar to that of PcrA alone (Figure 2B, purple *vs*. light blue line). Similar results were observed in the presence of 3.2 or 4.8 PcrAs/cssDNA molecule (Figure 2B, orange and dark green *vs*. light blue line). It is likely that: 1) *circa* one PcrA monomer per cssDNA is necessary to strip RecA without any obvious delay upon the addition to dynamic RecA-cssDNA complexes; and 2) PcrA loads on DNA secondary structures and disrupts and strips RecA nucleoprotein filaments from the cssDNA because PcrA binding at positions vacated by RecA·ADP should require a lag phase.

### PcrA K37A Neither Removes RecA Nor Stimulates Its ATPase Activity

A PcrA_*Sau*_ mutant variant (PcrA_*Sau*_ K33A Q250R), which fails to hydrolyze ATP and unwind DNA, facilitates the displacement of RecA_*Eco*_ from linear dT_21_ ssDNA (Anand et al., 2007). However, RecA_*Eco*_, which forms unstable complexes on dT_21_ ssDNA (Joo et al., 2006), may undergo disassembly from unstructured ssDNA due to any small disturbance (*e.g.*, altering the buffering condition of the reaction mixture upon PcrA_*Sau*_ K33A Q250R addition). To test whether the ATPase activity of PcrA was necessary to displace a growing RecA filament or if PcrA displaces RecA by stimulating its ATPase activity, a PcrA Walker A box mutant (PcrA K37A [counterpart of PcrA_*Sau*_ K33A]), which poorly hydrolyses ATP, was purified. In the presence of plasmid-size natural cssDNA, the PcrA K37A mutant variant shows a poor ATPase activity, ∼60-fold lower (K_cat_ of 26.5 ± 5.0 min^−1^) than that of *wt* PcrA (Figure 2A, dark blue line and Supplementary Figure S2B, orange line). Similar results were previously reported with PcrA_*Gst*_ K37A (Soultanas et al., 1999).

PcrA K37A does not affect the ATPase activity of RecA filamented on plasmid-size cssDNA. The ATPase activity of pre-formed RecA-cssDNA complexes, even in the presence of ∼10 PcrA K37A monomers/cssDNA molecule (1 PcrA K37A/333 nt), reached a steady-state rate of ATP hydrolysis with a slope comparable to that of RecA alone (Supplementary Figure S2B, green *vs.* light blue line). It is likely that: 1) PcrA uses ATP hydrolysis to translocate on the ssDNA; and 2) PcrA K37A neither passively nor actively displaces RecA from plasmid-size cssDNA within the 20 min reaction (Supplementary Figure S2B). Similarly, UvrD_*Eco*_ K35I, which fails to hydrolyze ATP, does not facilitate RecA_*Eco*_ disassembly from plasmid-size cssDNA (Petrova et al., 2015).

### PcrA Strips RecA Filaments but Not an Unrelated Enzyme Translocating in the 5′→3′ Direction

In a previous section, we have shown that *circa* one PcrA monomer/cssDNA molecule can strip RecA from cssDNA (Figures 2A,B). Previously, it has been shown that: 1) a PcrA monomer translocates in a processive manner along ssDNA with a speed of ∼270 nt/s, but multiple monomers translocate stepwise along ssDNA and unwind the duplex at a speed of ∼50 bp/s in the 3′→5′ direction (Niedziela-Majka et al., 2007; Singleton et al., 2007; Lohman et al., 2008); 2) RecA_*Eco*_ nucleated and filamented onto a 24 nt or longer ssDNA region is competent for ATP hydrolysis (Sussman et al., 2008; Bell and Kowalczykowski, 2016); and 3) UvrD_*Eco*_ drives fork processing and indirectly inhibits RecA_*Eco*_-mediated remodeling (Flores et al., 2004). To test whether PcrA translocates and unwinds one or both strands (fork remodeling), we used a HJ-like structure having one 30 nt long 3′-arm (a 3′-ssDNA tail HJ DNA), as depicted in Supplementary Figure S3. The 30 nt ssDNA of the tail HJ DNA can accommodate up to 4 PcrA molecules (see above).

PcrA (0.35–12 nM) was incubated with the 3′-tailed HJ DNA substrate (0.5 nM in molecules), and translocation and unwinding experiments were performed. In the presence of a limiting PcrA concentration (∼0.7 and ∼1.5 PcrAs/3′-tailed HJ DNA molecule), the 3′-tailed HJ substrate was not significantly unwound (Supplementary Figure S3, lanes 2-3). As PcrA concentration increased, the 3′-tailed HJ substrate was unwound in the 3′→5′ direction, yielding a three-way junction and forked DNA in the presence of ∼4 PcrAs/3′-tailed HJ DNA molecule (Supplementary Figure S3, lanes 4-5). This is consistent with the observation that PcrA binds ssDNA with a K_Dapp_ of ∼1.5 nM (Moreno-del Álamo et al., 2021). At higher PcrA concentrations, PcrA utilized the energy derived from ATP hydrolysis to translocate along the 3′-tail up to the duplex region and fully unwind the 3′-tailed HJ DNA, rendering the accumulation of the radiolabeled strand (Supplementary Figure S3, lanes 5–7). In the presence of ∼24 PcrA monomers/DNA molecule, all the 3′-tail HJ DNA substrate was unwound to yield the labeled strand (Supplementary Figure S3, lane 7), suggesting that the displaced arms might titer PcrA. Based on the available literature (Niedziela-Majka et al., 2007; Singleton et al., 2007; Torres et al., 2021), it is likely that PcrA, bound to the 30 nt tail of the HJ substrate, couples ATP hydrolysis to unwind one strand by step, but it cannot produce the force necessary to branch migrate the HJ DNA substrate to obtain forked DNA, as observed with a genuine translocase (*e*.*g*., RecG) (Kowalczykowski, 2015). Our data do not support the hypothesis that PcrA promotes fork remodeling, as suggested for UvrD_*Eco*_ (Flores et al., 2004).

To test whether PcrA translocating on a short ssDNA region strips RecA polymerizing on it, the 3′-ssDNA tail HJ DNA was pre-incubated with a large excess of RecA (400 nM), and then increasing PcrA concentrations were added. The presence of ∼4 PcrAs/3′-tailed HJ DNA molecule was necessary to displace RecA and unwind ∼50% of the 3′-tailed HJ substrate (Supplementary Figure S3, lanes 2–7 *vs.* lanes 9–14), suggesting that PcrA efficiently unwinds the 3′-tailed HJ DNA substrate independently of the presence or absence of RecA. It is likely that more than two PcrA molecules strip RecA from the 3′-tailed HJ DNA substrate and then unwind the DNA substrate. To demonstrate that RecA was filamented on the 3′-tailed HJ substrate, we took advantage of the observation that RecA filamented on a 3′-fork DNA substrate is necessary and sufficient to activate hexameric RadA/Sms to catalyze the unwinding of the complementary strand by moving in the 5′→3′ direction (Torres et al., 2019; Torres and Alonso, 2021). RadA/Sms did not significantly unwind the substrate, while RecA bound to the 3′-tail of the DNA substrate activates RadA/Sms to efficiently unwind the strand complementary to the 3′-tailed HJ DNA (Supplementary Figure S3, lane 18 *vs*. lane 19), confirming that RecA filamented on the 3′-tail of the HJ DNA substrate. It is likely that: 1) saturating RecA concentrations cannot compete PcrA for binding to ssDNA; 2) PcrA strips the RecA filament formed on the 3′-ssDNA tail of the HJ substrate before unwinding the HJ substrate; and 3) PcrA, by removing RecA from the 3′-tailed HJ DNA, may inhibit RadA/Sms loading and unwinding of the nascent lagging strand of a stalled fork with a gap in the template lagging-strand to facilitate RecG-mediated fork remodeling (Torres and Alonso, 2021; Torres et al., 2021).

**FIGURE 3:**
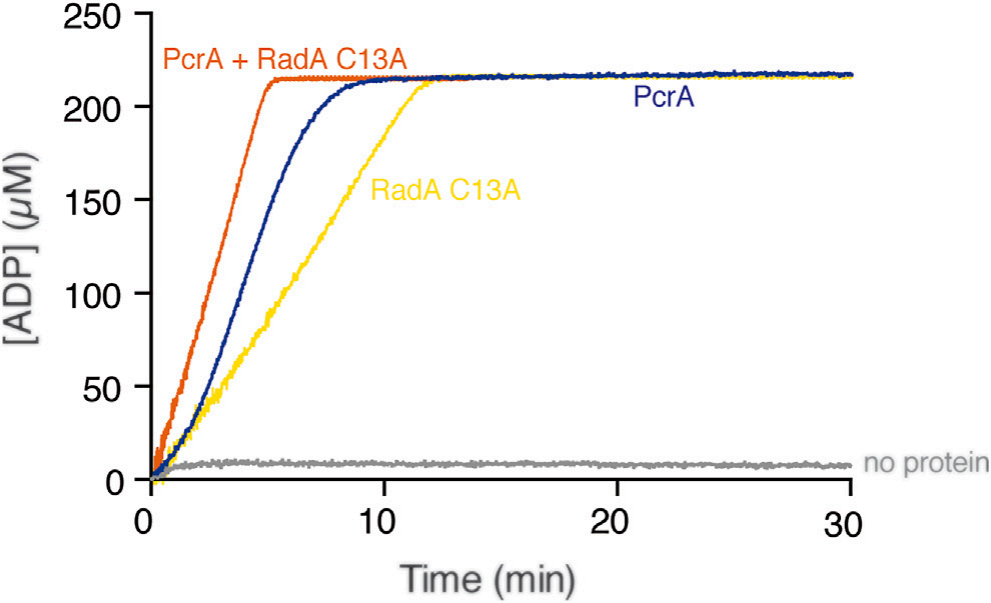
PcrA does not strip the RadA/Sms C13A ATPase. The cssDNA (10 μM in nts) was incubated with PcrA (15 nM), RadA/Sms C13A (33 nM), or both (5 min, 37°C) in buffer B containing 5 mM ATP and the NADH enzyme mix, and the ATPase activity was measured (30 min, 37°C). The grey line denotes the control reaction corresponding to the ATPase assay in the absence of any protein added.

Can PcrA dislodge any protein-DNA complex? To test whether PcrA translocating along cssDNA collides with and displaces any protein growing or moving in the opposite direction, we used the hexameric RadA/Sms C13A enzyme that binds cssDNA with ∼2-fold lower affinity than PcrA (Moreno-del Álamo et al., 2021; Torres et al., 2019). ATP hydrolysis-fueled RadA/Sms C13A translocation in the 5′→3′ direction reached the steady-state rate of ATP hydrolysis with a K_cat_ of 49.1 ± 1.5 min^−1^ (Figure 3, yellow line) (Torres et al., 2019). Under the buffer conditions optimal for RadA/Sms, PcrA hydrolyzed ATP with a K_cat_ of 1715 ± 305 min^−1^ (Figure 3, blue line). When both RadA/Sms C13A (∼10 RadA/Sms C13A hexamer/cssDNA molecule) and PcrA (∼5 PcrAs/cssDNA molecule) were incubated together with cssDNA, the maximal rate of ATP hydrolysis increased and approached the sum of their independent activities (K_cat_ of 1793 ± 202 min^−1^) (Figure 3, orange line). It is likely that: 1) both monomeric PcrA and hexameric RadA/Sms C13A bound to cssDNA moving in the opposite direction may dissociate with similar efficiency when undergoing head-on collisions; and 2) PcrA iterative motion on cssDNA is not sufficient to prevent re-loading of RadA/Sms C13A on cssDNA.

### SsbA Inhibits the ssDNA-Dependent ATPase Activity of PcrA

Previously, it has been shown that: 1) RPA_*Sce*_ interacts with and loads Srs2_*Sce*_ on the ssDNA (Kaniecki et al., 2017), but SsbA is not detected among the proteins that interact with PcrA (Costes et al., 2010); and 2) SsbA binds ssDNA with an average site size of ∼50 nt (mainly in the SSB_65_ and SSB_35_ binding modes) and with ∼7-fold higher affinity than PcrA, which binds ssDNA with a site size of 8 nt (Yadav et al., 2012; Moreno-del Álamo et al., 2021). To study the fate of PcrA recruitment on the SsbA-ssDNA complexes, a fixed PcrA (15 nM) and cssDNA were incubated with increasing SsbA concentrations (75–300 nM SsbA), and the maximal rate of ATP hydrolysis rate was monitored. We can hypothesize that: 1) the ATP hydrolysis rate of PcrA increases when it is loaded at ssDNA sites coated by SsbA; 2) PcrA loading is not compromised by SsbA, but PcrA efficiently displaces SsbA from ssDNA without altering the ATP hydrolysis rate; 3) PcrA loads at secondary structures formed on cssDNA, but SsbA compromises PcrA translocation on cssDNA; or 4) SsbA outcompetes PcrA for binding to cssDNA compromising the ATPase of PcrA.

We confirmed no ATP hydrolysis activity in the SsbA preparation (Figure 2C, orange line). In the presence of limiting SsbA (1 SsbA/133 nt, and at a SsbA:PcrA stoichiometry of 5:1), PcrA-mediated ATP hydrolysis was reduced ∼3-fold (K_cat_ of 450 ± 118 min^−1^) when compared with PcrA alone (Figure 2C, green *vs*. dark blue line). As the SsbA concentration increased, the ATP hydrolysis rate of PcrA decreased. In the presence of SsbA at a 20:1 stoichiometry relative to PcrA, and at 1 SsbA tetramer/33 nt of the ssDNA, the maximal rate of ATP hydrolysis by PcrA was significantly inhibited, by ∼18-fold (K_cat_ of 83 ± 10 min^−1^) (Figure 2C, light blue *vs*. dark blue line). It is likely that a SsbA-cssDNA complex adopts a unique structure that perturbs the translocation of PcrA on cssDNA, or PcrA cannot provide an opposite force to displace tightly bound SsbA from cssDNA.

RecA·ATP cannot nucleate on the SsbA-cssDNA complexes (K_cat_ of <1 min^−1^) (Figure 2D, red line) (Carrasco et al., 2015). In the presence of increasing SsbA and fixed RecA (800 nM) and PcrA (15 nM) concentrations, a steady-state rate of ATP hydrolysis with a slope comparable to that in the absence of RecA was observed (Figure 2D, green, brown, and light blue *vs*. 2C, green, brown, and light blue lines). It is likely that: 1) *circa* 5 PcrAs/cssDNA molecule dislodges RecA and partially redistributes tightly bound SsbA from cssDNA; and 2) SsbA-cssDNA complexes may compete PcrA for ssDNA binding rather than forming a structure that perturbs the translocation of PcrA on cssDNA. Different results, however, were observed with related enzymes. From one side, Srs2_*Sce*_ translocating in the 3′→5′ direction strips both Rad51_*Sce*_ and RPA_*Sce*_ from ssDNA (Qiu et al., 2013; Kaniecki et al., 2017). From another side, the total ATP hydrolysis rate is significantly reduced in the presence of ∼25 UvrD_*Eco*_ monomers/cssDNA molecule when incubated with pre-formed RecA_*Eco*_-cssDNA-SSB_*Eco*_ complexes, but the maximal rate of ATP hydrolysis is significantly increased, after a ∼25 min lag phase, in the presence of ∼50 UvrD_*Eco*_/ssDNA molecule when incubated with pre-formed RecA_*Eco*_-cssDNA-SSB_*Eco*_ complexes (Petrova et al., 2015).

### RecO Partially Antagonizes SsbA on the Inhibition of the ATPase of PcrA

*In vitro*, RecA neither nucleates on the SsbA-ssDNA complexes nor displaces SsbA by further binding onto ssDNA in the presence of ATP (K_cat_ of <1 min^−1^) (Figures 2D,E, red and orange lines) (Yadav et al., 2014). RecO (counterpart of eukaryotic Rad52) interacts with and partially displaces SsbA. The two-component mediator (SsbA and RecO) accelerates assembly of RecA filaments on ssDNA with a K_cat_ of 17.4 ± 1.1 min^−1^, when compared to RecA spontaneous growth rate (K_cat_ of 9.5 ± 1.9 min^−1^) (Figure 2E, orange and light blue *vs*. red line) (Carrasco et al., 2015). Here, RecA nucleation and subsequent filament formation was biphasic, with a slow nucleation step (∼5 min lag phase) (Figures 2E,F), as previously reported (Carrasco et al., 2015).

RecO is not detected among the Tap-tag proteins with PcrA (Costes et al., 2010). PcrA-mediated ATP hydrolysis was not affected by the presence of the positive RecO mediator (1 RecO/100 nt) (Supplementary Figure S5, blue *vs*. red line), suggesting that PcrA utilizes the energy derived from ATP hydrolysis to translocate along ssDNA and actively displace RecO. Similarly, neither Rad52_*Sce*_ nor Rad55_*Sce*_-Rad57_*Sce*_ affect Srs2_*Sce*_ translocation on ssDNA (Liu et al., 2011; De Tullio et al., 2017; Roy et al., 2021).

To test whether RecO displaces SsbA and indirectly contributes to stimulating the ATPase activity of PcrA, ATP hydrolysis assays were performed. The rate of ATP hydrolysis by PcrA was similar when incubated with a pre-formed SsbA-cssDNA or SsbA-cssDNA-RecO complex (Supplementary Figure S4). It is likely that: 1) PcrA is not loaded at SsbA-ssDNA regions because PcrA-mediated ATP hydrolysis was similar in the presence of SsbA or SsbA and RecO; and 2) PcrA dismantles RecO but poorly removes SsbA from cssDNA.

When the two-component mediator (SsbA and RecO) was pre-incubated with cssDNA and then PcrA and RecA were added, the maximal rate of ATP hydrolysis was reached without any lag time. The final state rate of ATP hydrolysis was significantly increased (K_cat_ of 105 ± 34 min^−1^) when compared to that of RecA-cssDNA (Figure 2E, green line) or SsbA-cssDNA-PcrA complexes (Figures 2C,D), but it did not reach the levels as with PcrA alone (4.8 PcrAs/cssDNA molecule) (Figure 2E, dark blue line). To assess the contribution of SsbA and RecO to both ATPases, PcrA was added to pre-formed SsbA, RecO, RecA, and cssDNA quaternary complexes (Figure 2F). Stoichiometric SsbA and limiting RecO and RecA concentrations hydrolyzed ATP at near the previously observed K_cat_ (17.0 ± 1.2 min^−1^) (Figure 2F, red line). Ten minutes later, increasing PcrA concentrations were added to the quaternary complexes. In the presence of 1.6 PcrAs/cssDNA molecule, the maximal rate of ATP hydrolysis was marginally affected with respect to RecA alone (Figure 2F, purple *vs*. red line). In the presence of 3.2 PcrAs/cssDNA molecule, the steady-state rate of ATP hydrolysis showed a slope comparable to RecA (Figure 2F, orange line). As the enzyme concentration increases (4.8 PcrAs/cssDNA molecule), PcrA utilized the energy derived from ATP hydrolysis to partially displace SsbA and to strip both RecO and RecA without a lag time, and the steady-state rate of ATP hydrolysis showed a slope comparable to the one of PcrA alone (Figure 2F, green *vs*. dark blue line). It is likely that: 1) RecO and SsbA do not inhibit PcrA translocation on the cssDNA; 2) RecO and SsbA counterbalance the strippase activity of PcrA, *circa* 3 PcrA molecules translocating on cssDNA strip RecA, and SsbA and RecO accelerate reassembly of a RecA filament on ssDNA; and 3) *circa* 5 PcrA molecules exhibit an ATP-dependent striping activity to prevent RecA reassembly, and RecO and SsbA are not sufficient to reverse PcrA-mediated dismantling of RecA filaments, suggesting that other player(s) may contribute to stabilizing RecA filaments. Likewise, the activity of the PcrA ortholog Srs2_*Sce*_ is opposed by the Rad52_*Sce*_ or Rad55_*Sce*_-Rad55_*Sce*_ mediators (Burgess et al., 2009; Roy et al., 2021).

### PcrA Poorly Strips RecA·dATP From cssDNA

**FIGURE 4:**
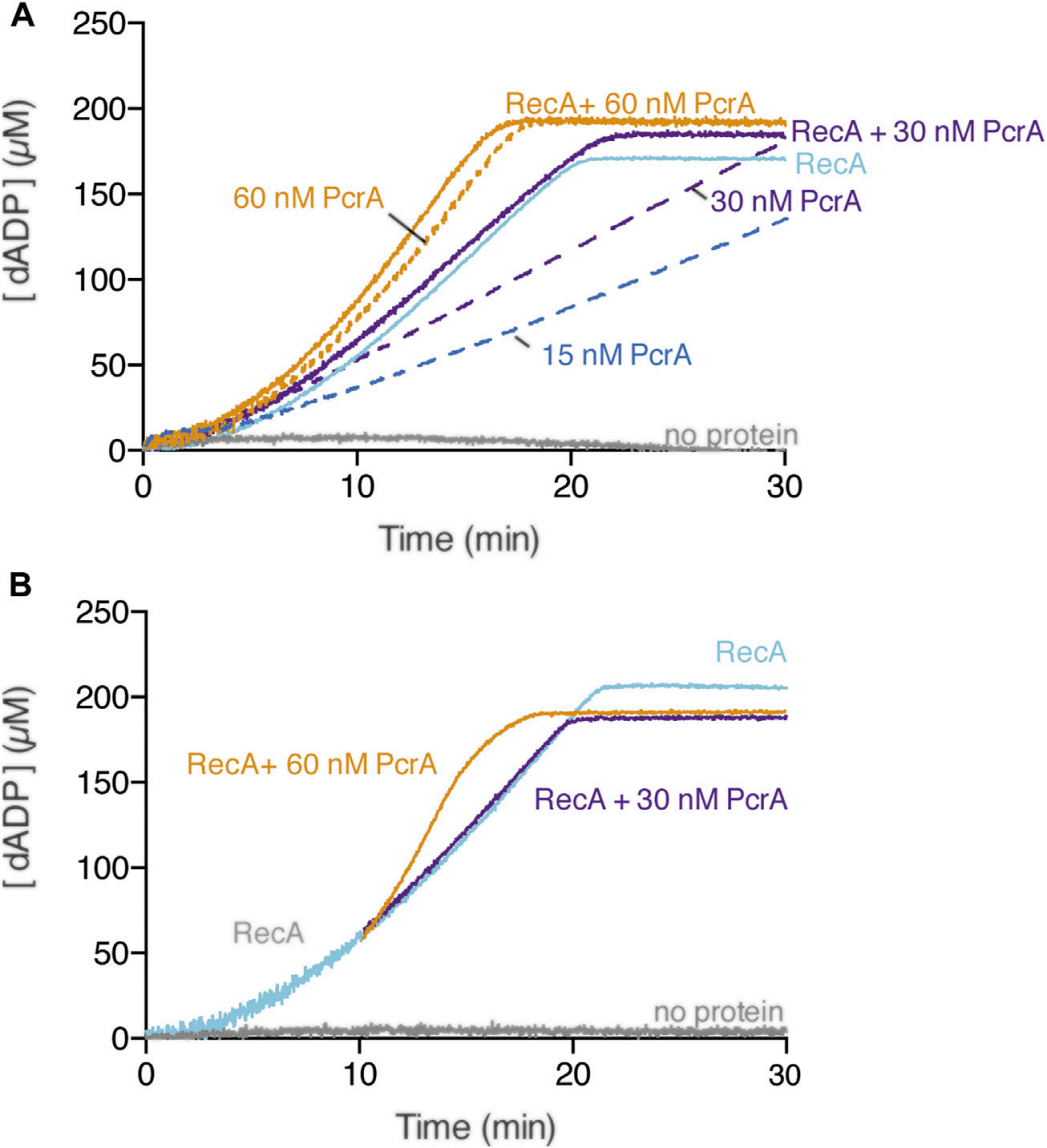
Multiple PcrAs inhibit RecA-mediated dATP hydrolysis. **(A)** PcrA (15–60 nM), RecA (800 nM), or both proteins were incubated with cssDNA (10 μM in nts) in buffer A containing 5 mM dATP **(A)**; or RecA and cssDNA were pre-incubated (10 min, 37°C) in buffer A containing 5 mM dATP, and then increasing PcrA concentrations were added **(B)**. The grey line denotes the control reaction corresponding to the dATPase assay in the absence of any protein, and the broken lines represent the presence of only PcrA at the indicated concentration **(A,B)**. The dATPase activity was measured (30 [or 20] min, 37°C). Representative graphs are shown here, and the determined K_cat_ is described in the text.

RecA in the dATP bound form is more effective than in the ATP bound form in nucleating on naked cssDNA, and only RecA·dATP can nucleate on the SsbA-cssDNA complexes (Lovett and Roberts, 1985; Carrasco et al., 2008; Manfredi et al., 2008; Steffen and Bryant, 1999). RecA·dATP (800 nM) nucleates with a lag time of 4 ± 0.5 min and efficiently polymerizes onto cssDNA at near the previously observed k_cat_ of 18.2 ± 0.4 min^−1^ (Figure 4A, light blue line) (Yadav et al., 2014). PcrA, however, hydrolyses dATP with lower efficiency than ATP (Singleton et al., 2007). In the presence of *circa* 5 PcrA monomers/cssDNA molecule (1 PcrA/666 nt), PcrA-mediated dATP hydrolysis showed a ∼3 min lag phase. Then, the maximal dATP hydrolysis rate with a K_cat_ of 605 ± 80 min^−1^ was reached (Figure 4A, blue broken-lines) (Moreno-del Álamo et al., 2021). Similar results were observed in the presence of *circa* 10 or 20 PcrA monomers/cssDNA molecule (Figure 4A, purple and orange broken lines). To test whether PcrA displaces a more dynamic RecA·dATP nucleoprotein filament, ATP was replaced by dATP, and the dATP hydrolysis rate was measured.

In the presence of ∼10 PcrAs/cssDNA molecule, the rate of dATP hydrolysis did not increase above that seen with RecA alone (Figure 4A, purple line). As PcrA concentration increased, the maximal rate of dATP hydrolysis increased above that seen with RecA alone. An array of ∼20 PcrAs/cssDNA molecule is necessary to reach an dATP hydrolysis rate with a slope similar to that on isolated circular ssDNA (Figure 4A, orange *vs*. broken orange line).

How can we explain the discrepancies in the presence of ATP *vs*. dATP? Since RecA·dATP cooperative filament growth shows a higher redistribution (k_cat_ of 18.2 ± 0.4 min^−1^) when compared with RecA·ATP (k_cat_ 9.5 ± 1.9 min^−1^) (Figures 2A, 3A, light blue line) and PcrA shows a lower iterative translocation because a significant higher PcrA concentration, by ∼12-fold, is required to displace filamented RecA·dATP when compared to RecA·ATP, we assumed that PcrA does not stimulate the ATPase activity of RecA when cleared by PcrA from cssDNA.

### Multiple PcrA Molecules Inhibit RecA-Mediated DNA Strand Exchange

Previously, it has been shown that: 1) a two-component mediator (RecO-SsbA) is necessary and sufficient to activate RecA·ATP to catalyze DSE (Carrasco et al., 2015; Carrasco et al., 2016), but RecA·dATP catalyzes DSE in the absence of mediators (Lovett and Roberts, 1985; Steffen and Bryant, 1999); and 2) monomeric PcrA is a highly processive 3′→5′ ssDNA translocase that binds to and moves along ssDNA with a speed of ∼270 nt/s but stalls at a duplex junction, and multiple PcrA molecules are needed to unwind dsDNA with a speed of ∼50 bp/s in the 3′→5′ direction (Niedziela-Majka et al., 2007; Singleton et al., 2007).

**FIGURE 5:**
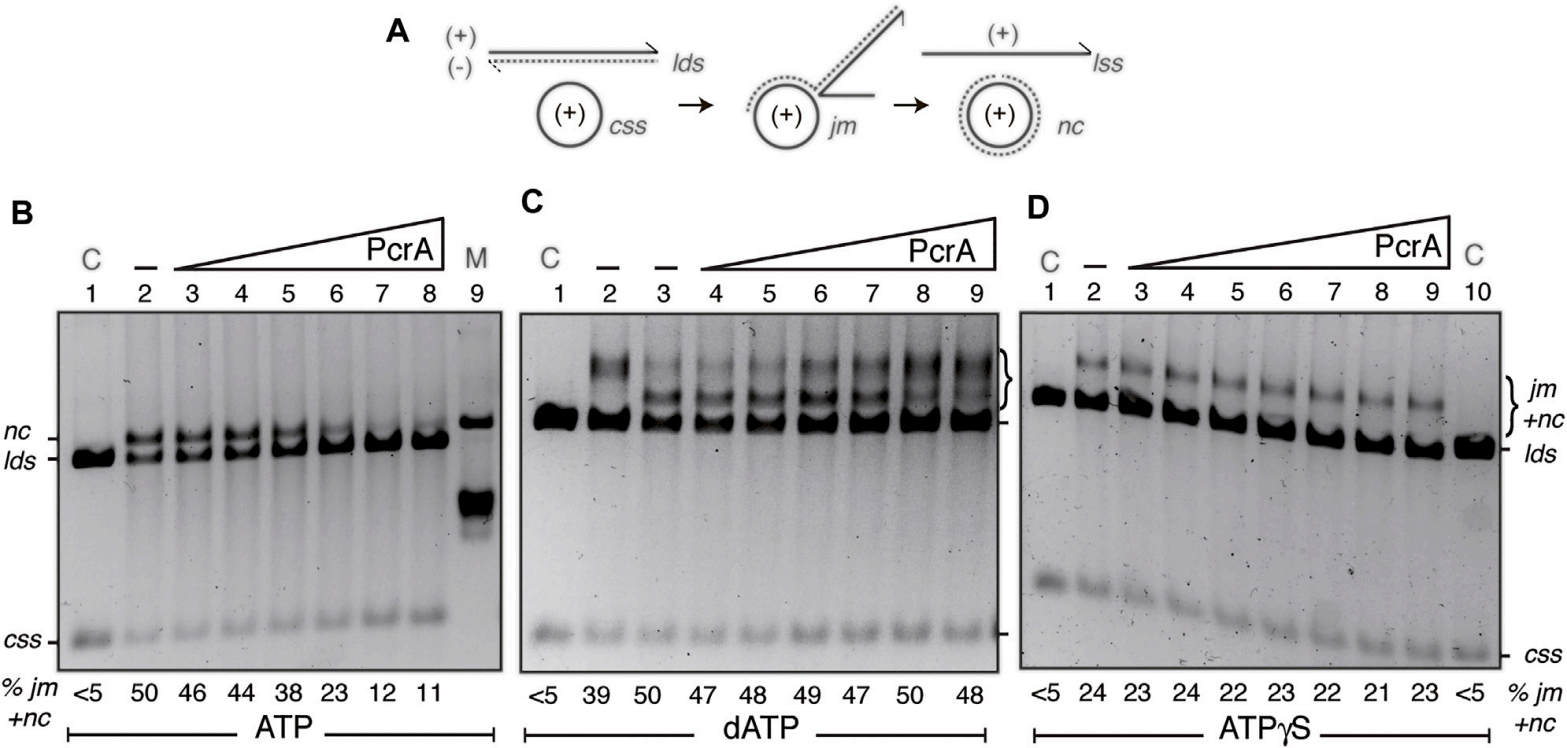
PcrA inhibits RecA-mediated DSE. **(A)** Cartoons illustrating the substrates, intermediates, and products of the three-strand exchange reaction upon RecA-mediated DSE. **(B,D)** The cssDNA (10 μM in nt) was pre-incubated with SsbA (300 nM) and RecO (100 nM) in buffer A (for 5 min at 37°C). Then, increasing PcrA (3–100 nM) [B-D]) and fixed RecA (800 nM), *lds* (10 μM in bp), and ATP **(B)** or ATPγS **(D)** (5 mM) concentrations were added, and the reaction was incubated for 60 min at 37°C. **(C)** The cssDNA was incubated with increasing PcrA (1.5–100 nM), fixed RecA, *lds*, and dATP (5 mM) concentrations for 60 min at 37°C. The products of the reactions were deproteinized, separated on a 0.8% AGE with ethidium bromide, and quantified as described in Materials and Methods. The positions of the bands corresponding to *css*, *cds*, *lds*, *jm, nc*, and ATPγS-generated *prd* products are indicated. Symbols - denote the absence of PcrA. In **(B,D)**, lanes 1 and 10, the cssDNA and the *lds* substrates are denoted as control **(C),** and in A (lane 9), nicked circular pGEM3 Zf (+) plasmid DNA was added as a mobility control of the *nc* product. The % of *jm*s and products (*nc* and *prd*) are indicated and expressed as the percentage of total substrate added. The results are the average value obtained from more than three independent experiments, and representative gels are shown here (the results given stand within a 5% standard error).

RecA-mediated DSE is a multistep reaction. 1) SsbA efficiently binds to cssDNA, RecO interacts with SsbA altering the SsbA-cssDNA complex to facilitate RecA nucleation, and the stabilized RecA promotes filament growth with a partial displacement of SsbA (activated RecA) (Yadav et al., 2012; Carrasco et al., 2015). 2) A dynamic RecA filament [with the help of the two-component mediator (SsbA and RecO)] catalyzes homology search; once a region of homology is found, the RecA-ssDNA filament catalyzes pairing with dsDNA at the three-stranded displacement loop (D-loop) to form a joint molecule (*jm*) intermediate. Here, RecA binds the complementary strand, and its secondary binding site interacts with the non-complementary strand (Chen et al., 2008; Yang et al., 2020). 3) RecA bound to the D-loop catalyzes strand transfer, with SsbA bound to the displaced strand to stabilize the *jm* and generate nicked circular (*nc*) and linear ssDNA products (forward DSE reaction) (Chen et al., 2008; Yang et al., 2020). To test whether PcrA inhibits RecA-mediated DSE by stripping RecA from the cssDNA (stage [1]) or at any other stage (2, 3), the three-strand exchange reaction was performed in the presence of SsbA, RecO, and ATP or ATPγS as a nucleotide cofactor (see above), and in the absence of mediators in the presence of dATP (Figures 5A–D).

First, the fixed 3199 nt cssDNA (10 μM in nt) and a homologous linear 3199 bp (*lds*) DNA (10 μM in bp) were incubated with RecO (1 RecO/100 nt), RecA (1 RecA/12.5 nt), stoichiometric SsbA (1 SsbA/33 nt), and increasing PcrA concentrations (3–100 nM) in buffer A containing 5 mM ATP for 60 min at 37°C (Figure 5B). In the absence of PcrA, RecA efficiently catalyzed the formation of a *jm* intermediate, and then ∼50% of the linear duplex (*lds*) and cssDNA (*css)* substrates were converted onto the *nc* and the linear ssDNA products, but no spontaneous DSE was observed in the absence of the proteins (Figure 5B, line 2 *vs*. line 1). In the presence of 1–4 PcrA monomers/cssDNA molecule, RecA efficiently catalyzed the formation of a *jm* intermediate and a *nc* product (Figure 5B, lines 3–5 *vs*. line 2), suggesting that stages 1–3 should not be inhibited, and PcrA disruption is overcome by SsbA and RecO by accelerating RecA reassembly. As PcrA concentration increases, RecA-mediated DSE was reduced by ∼2-fold in the presence of 8 PcrAs/cssDNA molecule (at a 1:32 PcrA:RecA stoichiometry) (Figure 5B, line 6 *vs*. line 2) and by ∼4-fold in the presence of 16 PcrAs/cssDNA molecule (Figure 5B, line 7 *vs*. line 2). No further inhibition was observed by increasing PcrA concentrations (at a 1:8 PcrA:RecA stoichiometry) (Figure 5B, lane 8 *vs*. lane 7). It is likely that: 1) few reassembled RecA monomers bound to the cssDNA may be sufficient for homology search and DNA strand pairing, and the defect should be at stages (2) or (3) because *circa* 5 PcrA monomers/cssDNA molecule are sufficient to catalytically antagonize SsbA- and RecO-mediated RecA nucleation (Figures 2E,F, green line); and 2) multiple PcrA molecules may be required to inhibit RecA-mediated DSE in the presence of RecO and SsbA.

Second, to learn about the contribution of the mediators, we performed three-strand exchange reactions in the presence of dATP without the mediators. As previously described, RecA·dATP catalyzed DSE more slowly than in the presence of mediators (Yadav et al., 2012). About 40% of *lds* and the complementary *css* DNA were converted onto *jm* intermediates at 20 min, and ∼50% of the substrates were converted to *nc* products (Figure 5C, lines 2-3). The accumulation of the *jm* + *nc* product was not significantly affected in the presence of 1–8 PcrA monomers/cssDNA molecule (Figure 5C, lines 4–7 *vs*. line 3), but higher PcrA concentrations impair RecA·dATP-mediated accumulation of *nc* products (Figure 5C, lanes 8-9). It is likely that a more stable RecA·dATP-cssDNA complex is less sensitive to PcrA action, but ∼32 PcrA monomers/cssDNA molecule inhibit the conversion of *jm* intermediates onto *nc* recombinant products. Since the K_ms_ of RecA for both nucleotides are similar but the dATP pool in the cytosol is 100–500-fold lower than that of ATP (Yadav et al., 2014), it is likely that dATP may have a small but significant contribution to limit PcrA activities in RecA-mediated DSE.

Third, *in vitro*, PcrA binds ssDNA in the apo or ATP bound form, and in the presence of ATP, the enzyme utilizes the energy derived from ATP hydrolysis for translocating on ssDNA and unwinding duplex DNA (Singleton et al., 2007; Lohman et al., 2008). RecA requires ATP binding but not hydrolysis for nucleation, homology search, DNA strand invasion, and forward DSE. Inverse DSE does not occur with ATPγS (Cox, 2007; Kowalczykowski, 2015). To test whether PcrA pre-incubated with the poorly hydrolysable ATPγS analog affects RecA filament growth, perhaps by a roadblock capping mechanism, and indirectly reduces RecA-mediated DSE, ATP was replaced by ATPγS. RecA·ATPγS, in the presence of the two-component mediator, catalyzed DSE between the *lds* and *css* substrate to render a “*nc*” product that runs more slowly than a genuine *nc* product (Figure 5D, line 2 *vs*. line 1), as earlier reported (see Carrasco et al., 2015). PcrA concentrations as high as 100 nM (1 PcrA/100 nt) were not sufficient to impair RecA·ATPγS-mediated accumulation of final “*nc*” products (Figure 5D, lane 9). It is likely that PcrA bound to ssDNA cannot cap RecA filament growth and indirectly inhibit RecA-mediated DSE. However, we cannot rule out that RecA-mediated DSE in the 3´→5′ direction (see Carrasco et al., 2016) could mask the putative capping activity of PcrA. We wonder if the PcrA unwinding activity may be limited when RecA interacts with the displaced strand at a D-loop intermediate or PcrA has more than one activity to exert its inhibitory effect by competing with RecA for ssDNA binding sites.

### PcrA Does Not Work as a Cap of RecA Filament Growth

**FIGURE 6:**
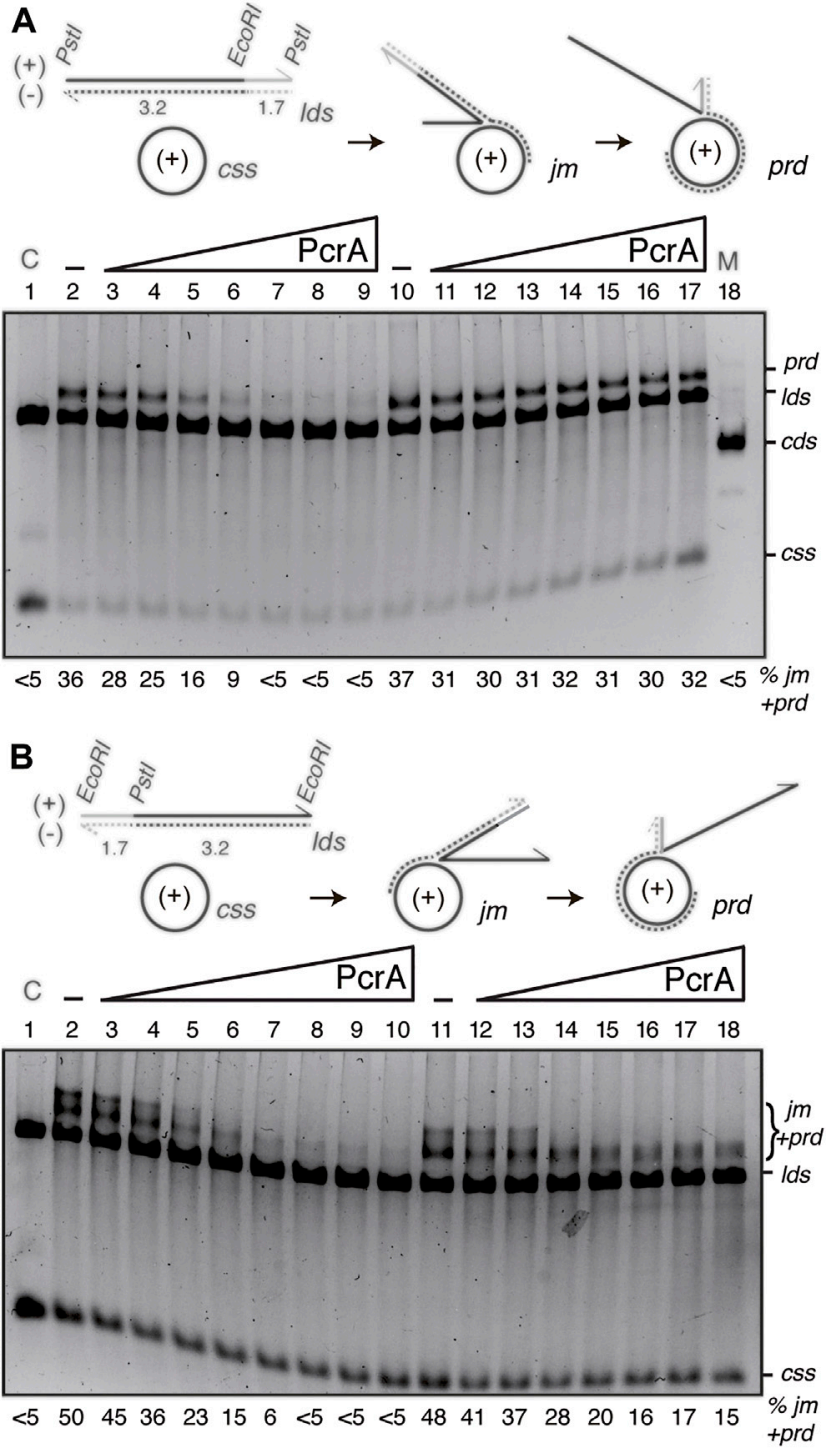
PcrA inhibits RecA-mediated DSE independently of the polarity of DSE. **(A,B)** Cartoons illustrating the three-strand exchange reaction between circular 3199 nt ssDNA (*css*, +) and the 4374 bp *lds* substrate with homology on the (-) strand restricted to the 3′-end (**(A)**, *Pst*I substrate) or to the 5′-end (**(B)**, *Eco*RI substrate). The expected *prd* final products of RecA-mediated DSE are illustrated. The relevant restriction sites are indicated. The relative lengths of homology (denoted in black) and heterology (denoted in grey) are indicated. (**(A,B)**, lanes 2–9) SsbA (300 nM) and RecO (100 nM) were pre-incubated with cssDNA (10 μM in nt) (for 5 min at 37°C). Then, increasing PcrA (6–400 nM) and fixed RecA (800 nM), *lds Pst*I **(A)** or *Eco*RI **(B)** (10 μM in bp) and ATP (5 mM) concentrations were added, and the reaction was incubated (60 min at 37°C). (**(A,B)**, lanes 10[11]–18[19]) SsbA (300 nM) and RecO (100 nM), RecA and *lds Pst*I **(A)** or *Eco*RI **(B)** were pre-incubated with cssDNA (10 μM in nt) (for 20 min at 37°C). Then, increasing PcrA (6–400 nM) and fixed ATP (5 mM) concentrations were added, and the reaction was incubated for 60 min at 37°C. The products of the reactions were processed as described in Figure 5. The positions of the bands and substrates are described in Figure 5. The % of *jm*s and products (*nc* and *prd*) are indicated and expressed as the percentage of total substrate added. The results are the average value obtained from more than three independent experiments and representative gels are shown here (the results given stand within a 5% standard error).

Previously, it has been proposed that PcrA_*Gst*_ in a head-on collision caps RecA_*Eco*_ filament growth in the 5′→3′ direction and passively favors its disruption from dT_40_ ssDNA (Fagerburg et al., 2012). It has been shown that RecA catalyzes bidirectional DSE, although RecA preferentially polymerizes at the 3′-end of the growing RecA filament (in the 5′→3′ direction) (Carrasco et al., 2016). To test whether PcrA by translocating in the 3′→5′ direction caps RecA filament growth, the three-strand exchange assay using substrates with heterology either at the 5′-end (a 4374 bp *lds* substrate containing a 1175 bp heterologous region at the 5′-end, *Pst*I-linearized substrate, Figure 6A) or at the 3′-end (the heterologous region at the 3′-end, *Eco*RI-linearized substrate) was performed (Figure 6B).

Limiting RecO and RecA, stoichiometric SsbA relative to cssDNA, and increasing PcrA concentrations were incubated with the *Pst*I- (Figure 6A) or *Eco*RI-linearized substrate (Figure 6B), and its complementary 3199 nt cssDNA substrate (+strand), in buffer A containing 5 mM ATP (60 min at 37°C). As the initial pairing reaction does not dissociate upon deproteinization (Carrasco et al., 2016), the three-stranded synaptic complexes occurring at either the 3′- or the 5′-complementary end were topologically interwound intermediates.

In the presence of RecO and SsbA, RecA·ATP filamented on the *css* substrate catalyzed DNA pairing and DSE with the duplex substrates (Figure 6). When the 3′-end of the (−) strand was homologous to the complementary *css* (+) substrate, RecA-mediated DSE displaced the 5′-end non-complementary (+) strand from duplex DNA in a 5′→3′ direction relative to the cssDNA to which RecA is initially bound, yielding a final recombination product (*prd*) (Figure 6A, lane 2), a *nc* product with a heterologous duplex tail (Carrasco et al., 2016). By contrast, when homology was at the 5′-end, RecA-mediated DSE displaced the 3′-end non-complementary (+) strand from duplex DNA in a 3′→5′ direction, yielding a high proportion of *jm* intermediates and recombinant *prd* (Figure 6B, lane 2).

The presence of ∼8 PcrA monomers/cssDNA was sufficient to reduce *prd* accumulation by ∼2-fold, and RecA-mediated DSE was inhibited at higher PcrA concentrations. About 16 PcrAs/ssDNA molecule were necessary to reduce *prd* accumulation by ∼6-fold independently of DSE polarity (Figures 6A,B, lane 5 *vs*. lanes 6–9). It is likely that: 1) PcrA inhibits RecA-mediated DSE independently of the polarity of the strand exchange reaction; and 2) a cluster of PcrA might be required to inhibit RecA-mediated DSE. Alternatively, PcrA stimulates the reverse reaction without affecting the forward RecA-mediated DSE.

Previously, it has been shown that: UvrD_*Eco*_ stimulates RecA-driven branch migration (Morel et al., 1993). To test whether PcrA stimulates the RecA-mediated strand exchange reverse reaction, fixed RecO, RecA, and SsbA concentrations were incubated with the DNAs for 20 min in buffer A containing 5 mM ATP, then increasing PcrA concentrations were added, and the reaction was further incubated for 40 min. In the presence of homology at the 3′-end, PcrA, at even a 1:2 PcrA:RecA molar ratio, was neither sufficient to reduce *prd* accumulation nor sufficient to reverse the reaction (Figure 6A, lane 10 vs. lanes 11–17). In the presence of homology at the 5′-end, 2–4 PcrA monomers/cssDNA allowed the conversion of *jm* into final *prd* products, but higher PcrA concentrations, even at a 1:2 PcrA:RecA molar ratio, were sufficient to reduce *prd* accumulation, but not to reverse the reaction (Figure 6B, lane 11 *vs*. lanes 12–17).

### PcrA Cannot Reverse RecA-Mediated DNA Strand Exchange

Previously, it has been shown that: 1) UvrD_*Eco*_ reverses RecA_*Eco*_-mediated *jm* formation (Morel et al., 1993); 2) the presence of sequence divergence halts RecA_*Eco*_-mediated branch migration, and the anti-recombinase UvrD_*Eco*_ reverses the recombination reaction upon interaction with MutSL_*Eco*_ bound to branch intermediates bearing a mismatch (Tham et al., 2013); and 3) RecA-mediated DSE is halted in the presence of an internal 77 bp region of heterology (54% sequence divergence) (Carrasco et al., 2019). To further evaluate whether PcrA bound to cssDNA reverses RecA-mediated DSE and if the translocase activity of PcrA assists RecA-mediated DSE to re-initiate beyond the region of heterology or from the 5′-distal end, specific DNA substrates were used. As described in Materials and Methods, two DNA substrates were chosen: 1) the *lds*_*het*_ DNA, which contains an internal 77 bp heterologous barrier (with identical dC:dG content, but with 42 mismatches) at position 424 from the 3′-end in an otherwise identical linear duplex substrate; and 2) the *lds*_*het-ins*_ DNA substrate, which contains one 77 bp heterologous region at position 424 and another at the 5′-end (Carrasco et al., 2019).

The *css*_*hom*_ and *lds*_*het*_ or *lds*_*het-ins*_ DNA substrates were incubated with fixed RecO, RecA, and SsbA and increasing PcrA concentrations (3–100 nM) in buffer A containing 5 mM ATP. When PcrA was omitted, RecA·ATP initiated DNA pairing at the homologous 3′-proximal end and trapped *jm* intermediates, with final recombination *nc* products barely detected with the *lds*_*het*_ DNA substrates and not detected with the *lds*_*het-ins*_ DNA substrates in a 60 min reaction (Supplementary Figure S5, lanes 2 and 11), as earlier documented (Carrasco et al., 2019). This is consistent with the observation that the heteroduplex cannot spontaneously branch migrate through the heterologous barrier, preventing RecA-mediated D-loop extension. However, recombination re-initiation from the 5′-distal end can occur for the *lds*_*het*_, albeit with low efficiency, while with the *lds*_*het-ins*_ substrate, no re-initiation is possible (see Carrasco et al., 2019). A sub-stoichiometric concentration of PcrA relative to ssDNA (1 PcrA/400 nt or at a 1:32 PcrA:RecA molar ratio) was sufficient to impair the accumulation of trapped *jm* intermediates with both DNA substrates, and at a 1:16 PcrA:RecA molar ratio, PcrA (1 PcrA/200 nt) inhibited RecA-mediated DSE (Supplementary Figure S5, lanes 6-7 and 15-16).

From the data presented in Figures 4–6, it can be inferred that there are two separable activities. First, up to 4 PcrA monomers/cssDNA molecule neither impair RecA-mediated *nc* product formation (Figure 5) or *prd* product formation (Figure 6) nor stimulate RecA bypass of the heterologous barrier (Figure S5). Second, multiple PcrA molecules (16 PcrA monomers/cssDNA) inhibit RecA-mediated *nc* product formation (Figures 5, 6) and block RecA-mediated bidirectional DSE (Supplementary Figure S5). We assumed that PcrA molecules, by unwinding the recombination intermediates, indirectly inhibit RecA-mediated DSE.

## Conclusion

We propose that *circa* one PcrA/cssDNA molecule (1 PcrA/2000 nt) catalytically displaces RecA from presynaptic filaments, and its iterative action prevents the reformation of nucleoprotein filaments on ssDNA when recombination is not needed. If recombination is needed, the RecA mediators, SsbA and RecO *in vitro* (or SsbA, RecO, and RecR *in vivo*), stimulate rapid RecA filament reassembly. A balance between these antagonic activities regulates RecA nucleoprotein filaments formation (Figure 2). However, it is poorly understood how PcrA is recruited to RecA-bound ssDNA, how SsbA and RecO tilt the balance against the PcrA anti-recombinase activity in RecA filament formation, and which other functions may contribute to antagonizing the anti-recombinase activity of PcrA. We show that, in the presence of ATP, a 10-fold excess of PcrA relative to cssDNA (1 PcrA/200 nt) is required to antagonize the branch migration phase of the RecA strand transfer reaction in the presence of positive mediators (Figure 5B), whereas, in the presence of dATP, a higher concentration of PcrA should be required to affect RecA-mediated DSE in the absence of positive mediators (Figure 5C). A significantly higher concentration of UvrD_*Eco*_ (1 UvrD_*Eco*_/10 nt) or PcrA_*Sau*_ (1 PcrA_*Sau*_ or PcrA_*Sau*_ K33A Q250R/11 nt) relative to cssDNA is required to inhibit RecA_*Eco*_-mediated DSE in the presence of ATP and absence of positive mediators (Veaute et al., 2005; Anand et al., 2007). It will be of significant interest to reconstitute the molecular mechanisms of recombination and define the subset of proteins required for the formation and regulation of an active RecA nucleoprotein filament able to perform homology search.

## Data Availability

The raw data supporting the conclusions of this article will be made available by the authors without undue reservation.
