# Supplementary material for: PcrA Dissociates RecA Filaments and the SsbA and RecO Mediators Counterbalance Such Activity
Source: Front Mol Biosci. 2022 Feb 9;9:836211. doi: 10.3389/fmolb.2022.836211 (PMC8865920; doi:10.3389/fmolb.2022.836211)
Supplement: Supplementary file 1 [file Presentation1.pdf]

PcrA dissociates RecA filaments and SsbA and RecO mediators counterbalance such activity

Begoña Carrasco†, María Moreno-del Álamo†, Rubén Torres and Juan Carlos Alonso*

Department of Microbial Biotechnology, Centro Nacional de Biotecnología, CNB-CSIC, 28049 Madrid, Spain

1. **Supplemental Annex 1.** PcrA depletion lethality is suppressed by *recO* or *recA* inactivation, but not by *mfd* inactivation

The interplay of PcrA with Mfd, RecO and RecA is poorly understood, and its analysis should help us to understand the primary contribution of PcrA to cell viability (1). When the *pcrA*$_T$ (*pcrA-ssrA sspB*) degron strain (see Material and methods) was grown in rich LB medium to $OD_{560} = 0.4$ (~5 x 10^7 CFUs/ml) and plated on plates containing IPTG, cell viability is reduced by >1000-fold when compared to the *wt* strain plated under similar conditions (Figure 1) (2, 3). Here, IPTG induces SspB expression. SspB bound to the SsrA moiety of PcrA-SsrA delivers the fused protein to the ClpXP protease for degradation (*pcrA*$_T$ strain), leading to PcrA depletion (2, 4, 5). Thus, as a first step to understand the primary cause of PcrA lethality, we re-analyzed the phenotype of PcrA depleted cells in the absence of RecO, RecA or Mfd, but a discrepancy with the previously reported results was observed. To unravel the unexpected discrepancies, the double (Δ*mfd pcrA*$_T$, *recO*16 *pcrA*$_T$, Δ*recA pcrA*$_T$) mutant strains were reconstructed by mobilizing the Δ*mfd*, *recO*16 or Δ*recA* mutation into the *pcrA*$_T$ (BG1525 strain) by SPP1-mediated generalized transduction. The newly constructed strains and those strains listed in Table S1 (*pcrA*$_T$ [BG1525], *recO*16 [BG107-1], Δ*recA* [BG1873], Δ*mfd* [BG1921], *recO*16 *pcrA*$_T$ [BG1715], Δ*recA pcrA*$_T$ [BG1877] and Δ*mfd pcrA*$_T$ [BG1875]) were grown in rich LB medium to $OD_{560} = 0.4$, and plated on plates containing 500 μM IPTG.

In the presence of IPTG, PcrA depletion lethality was suppressed by >1400-fold by *recO* or *recA* inactivation (Figure 1, yellow bars) as earlier described (1). Since the results with the newly constructed *recO pcrA*$_T$ or *recA pcrA*$_T$ were indistinguishable from the ones obtained with the *recO*16 *pcrA*$_T$ or Δ*recA pcrA*$_T$ strains listed in Table S1, the data of the previously reported strains (BG1877 and BG1715) were plotted in Figure 1 (yellow bars). Unexpectedly, when the newly constructed Δ*mfd pcrA*$_T$ (BG1923) strain and the old strain (BG1875) were analyzed, a different outcome was observed. PcrA depletion inviability did not require Mfd when the BG1923 strain was analyzed. The presence of the Δ*mdf* mutation in the BG1923 strain was confirmed by nucleotide sequencing. This contradicts our previous report in which PcrA depletion lethality and sensitivity to H_2O_2 was suppressed by *mfd* inactivation (BG1875 strain). There, the resulting colonies were minute and with an area ~19-fold smaller than in the absence of IPTG (1).

To understand this conflicting observation, we reconstructed again the strain by mobilizing the Δ*mfd* mutation, or the *pcrA*-*ssrA* and *sspB* cassettes by SPP1-mediated generalized transduction and/or chromosomal transformation into the *wt* BG214 strain. First, mobilization of the Δ*mfd* mutation on the *wt* strain revealed that the new Δ*mfd* clones were moderately sensitive to 4NQO, and as sensitive as the control Δ*mfd:Cm* or Δ*mfd:Em* strain (6, 7), independently of the mobilization route used. When the *pcrA-ssrA* or *sspB* cassettes were mobilized to obtain the $pcrA_T$ degron strain, upon addition of IPTG the cell viability was reduced by >1000-fold when compared to the *wt* strain plated under similar conditions. Finally, the Δ*mfd* mutation was mobilized into the $pcrA_T$ background. Several random Δ*mfd* $pcrA_T$ clones were selected for further analysis. PcrA depletion lethality between the different reconstituted Δ*mfd* $pcrA_T$ strains was not significantly different from the Δ*mfd* $pcrA_T$ (BG1923) strain, thus the information obtained with the BG1923 strain was plotted in Figure 1 (yellow bar). It is known that Mfd may function as an anti-mutator in DNA damage-induced mutagenesis, and it appears to function as a mutator for spontaneous mutagenesis (8, 9). It is likely that unselected mutations accumulated in the former Δ*mfd* $pcrA_T$ BG1875 strain (1), thus further work is required to understand the differences between the reported BG1875 and the newly constructed clones, represented by the BG1923 strain.

2. Supplemental Annex 2. PcrA transiently interacts RecA

Previously it has been shown that RecA, as well as the different subunits of RNAP, co-purifies with PcrA by Tap-tag experiments (10). To test whether PcrA physically interacts with RecA or such interaction is indirect, we analyzed the potential PcrA-RecA interaction by using immuno-dot blot assays (11). In short, increasing concentrations of PcrA (500 to 2000 ng) were applied to a pre-wetted nitrocellulose membrane as described in Materials and methods. In parallel, a similar concentration of BSA (as a negative) and RarA (as a positive control 12) were applied to the nitrocellulose membrane. The membrane was incubated with RecA (400 ng) and successively washed, then with polyclonal anti-RecA antibodies we highlight the presence of RecA. A 10-fold excess of PcrA retained RecA on the membrane (Figure S1A), and *circa* stoichiometric RarA concentrations were sufficient to retain RecA, but as expected even an excess of BSA was not sufficient to retain RecA under similar conditions (Figure S1A). It is likely that RecA physically interacts with PcrA, albeit such interaction may be weak. To confirm this result and to evaluate the strength of such protein-protein interactions, the retention of RecA by His-tagged PcrA bound in a Ni^{2+} matrix in the presence of increasing NaCl concentrations was tested as described (13). His-tagged PcrA (1 μg) and native RecA (1 μg) in a buffer condition at which the ATP hydrolysis assays are performed (buffer A) were separately incubated with 50 μl of the Ni^{2+} matrix and the flow-through (FT) was collected. The Ni^{2+} matrix was washed four times with 500 μl of buffer A (the first [W1] and the last [W6]) were collected) containing 20 mM imidazole, and eluted (E) with Buffer A containing 1 M NaCl and 0.4 M imidazole (Figure S1B). His-tagged PcrA (predicted mass 83.5 kDa) was retained on the 50-μl Ni^{2+} matrix, and after several washes the protein was eluted (E), but RecA (predicted mass 38.0 kDa) was present in the FT. No RecA protein was detected upon elution when the collected protein fractions were separated by 12.5% (w/v) SDS-PAGE (Figure S1B, lanes 6 and 10). Unexpectedly, when both His-tagged PcrA and RecA were pre-incubated (5 min at 37 ºC) and loaded into the Ni^{2+} column, we observed that RecA was only present in the FT in the presence of 50 mM NaCl, but PcrA was eluted with buffer A containing 1 M NaCl and 0.4 M imidazole (Figure S1B, lanes 13 *vs*. 16). Since His-tagged PcrA binds to ssDNA in buffer A (50 mM NaCl) with similar efficiency than PcrA upon removal of the

His-tag (13), and the maximal ATP hydrolysis rate of PcrA and His-tagged PcrA are similar and not affected by marginally increasing the ionic strength (80 mM NaCl) (Figure 1), we assume that the PcrA interaction with RecA has to be transient and weak.

3. **Table S1**.

Strains[a]	Relevant genotype	Source/reference
BG214	wild type	Laboratory strain
BG107-1	+ *recO*16	(1)
BG1873	+ Δ*recA*	(1)
BG1887	+ Δ*mfd*	(1)
BG339	+ Δ*mfd*, *cat*	(6)
BG1921	+ Δ*mfd*, *ermC*	This work
BG1525	+ *pcrA-ssrA sspB*	(3)
BG1715	+ *pcrA-ssrA sspB recO*16	(1)
BG1877	+ *pcrA-ssrA sspB* Δ*recA*	(1)
BG1875	+ *pcrA-ssrA sspB* Δ*mfd*	(1)
BG1923	+ *pcrA-ssrA sspB* Δ*mfd*	This work
**Plasmid**	**Relevant genotype**	**Source/reference**
pCB1229[b]	Amp^{R}, ori_{Eco} *pcrA*	(13)
pCB1230[b]	Amp^{R}, ori_{Eco} *pcrA* K37A	(13)
pCB722[c]	Amp^{R}, ori_{Eco} *ssbA*	(14)
pCB669[c]	Amp^{R}, ori_{Eco} *recO*	(15)
pCB1020[c]	Amp^{R}, ori_{Eco} *radA*	(16)
pCB1035[c]	Amp^{R}, ori_{Eco} *radA* C13A	(16)
pBT61[d]	Nn^{R}, ori_{Bsu} *recA*	(17)
pGEM3Zf(+)	Amp^{R}, ori_{Eco} ori_{f1}	Promega Biotech Ibérica

[a]All strains are derivatives of *B. subtilis* BG214 (*trpCE metA*5 *amyE1 ytsJ*1 *rsbV*37 *xre*1 *xkd*A1 *att*^{SPß} *att*$^{\text{ICE}Bs1}$) strain. [b]pCB1229 and pCB1230 were used to overexpress PcrA and PcrA K37A in the heterologous *E. coli* M15[pREP4] (QIAexpress) host. [c]pCB722, pBT669 and pCB1035 were used to overexpress SsbA, RecO and RadA/Sms C13A in the heterologous *E. coli* BL21(DE3)[pLysS] host. [d]pBT61 was used to overexpress RecA in the BG214 host.

4. References

1. M. Moreno-Del Alamo, R. Torres, C. Manfredi, J. A. Ruiz-Maso, G. Del Solar and J. C. Alonso: Bacillus subtilis PcrA Couples DNA Replication, Transcription, Recombination and Segregation. *Front Mol Biosci*, 7, 140 (2020) doi:10.3389/fmolb.2020.00140
2. C. N. Merrikh, B. J. Brewer and H. Merrikh: The B. subtilis Accessory Helicase PcrA Facilitates DNA Replication through Transcription Units. *PLoS Genet*, 11(6), e1005289 (2015) doi:10.1371/journal.pgen.1005289
3. R. Torres, H. Romero, V. Rodríguez-Cerrato and J. C. Alonso: Interplay between Bacillus subtilis RecD2 and the RecG or RuvAB helicase in recombinational repair. *DNA Repair (Amst)*, 55, 40-46 (2017) doi:10.1016/j.dnarep.2017.05.004
4. K. C. Keiler, P. R. Waller and R. T. Sauer: Role of a peptide tagging system in degradation of proteins synthesized from damaged messenger RNA. *Science*, 271(5251), 990-3 (1996) doi: 10.1126/science.271.5251.990

5. K. L. Griffith and A. D. Grossman: Inducible protein degradation in Bacillus subtilis using heterologous peptide tags and adaptor proteins to target substrates to the protease ClpXP. *Mol Microbiol*, 70(4), 1012-25 (2008) doi:10.1111/j.1365-2958.2008.06467.x
6. S. Ayora, F. Rojo, N. Ogasawara, S. Nakai and J. C. Alonso: The Mfd protein of Bacillus subtilis 168 is involved in both transcription-coupled DNA repair and DNA recombination. *J Mol Biol*, 256(2), 301-18 (1996) doi:10.1006/jmbi.1996.0087
7. J. M. Zalieckas, L. V. Wray, Jr., A. E. Ferson and S. H. Fisher: Transcription-repair coupling factor is involved in carbon catabolite repression of the Bacillus subtilis hut and gnt operons. *Mol Microbiol*, 27(5), 1031-8 (1998) doi:10.1046/j.1365-2958.1998.00751.x
8. E. M. Witkin: Mutation frequency decline revisited. *Bioessays*, 16(6), 437-44 (1994) doi:10.1002/bies.950160613
9. L. A. Lindsey-Boltz and A. Sancar: The Transcription-Repair Coupling Factor Mfd Prevents and Promotes Mutagenesis in a Context-Dependent Manner. *Front Mol Biosci*, 8, 668290 (2021) doi:10.3389/fmolb.2021.668290
10. A. Costes, F. Lecointe, S. McGovern, S. Quevillon-Cheruel and P. Polard: The C-Terminal domain of the bacterial SSB protein acts as a DNA maintenance hub at active chromosome replication forks. *PLoS Genet*, 6(12), e1001238 (2010) doi:10.1371/journal.pgen.1001238
11. B. W. Walsh, J. S. Lenhart, J. W. Schroeder and L. A. Simmons: Far Western blotting as a rapid and efficient method for detecting interactions between DNA replication and DNA repair proteins. *Methods Mol Biol*, 922, 161-8 (2012) doi:10.1007/978-1-62703-032-8_11
12. H. Romero, E. Serrano, R. Hernández-Tamayo, B. Carrasco, P. P. Cárdenas, S. Ayora, P. L. Graumann and J. C. Alonso: *Bacillus subtilis* RarA Acts as a Positive RecA Accessory Protein. *Front Microbiol*, 11, 92 (2020) doi:10.3389/fmicb.2020.00092
13. M. Moreno-del Alamo, B. T. Carrasco, R. and J. C. Alonso: *Bacillus subtilis* PcrA helicase removes trafficking barriers. *Cells*, 10, 935 (2021) doi:https://doi.org/10.3390/cells10040935
14. B. Carrasco, C. Manfredi, S. Ayora and J. C. Alonso: Bacillus subtilis SsbA and dATP regulate RecA nucleation onto single-stranded DNA. *DNA Repair (Amst)*, 7(6), 990-6 (2008) doi:S1568-7864(08)00127-4 [pii] 10.1016/j.dnarep.2008.03.019
15. C. Manfredi, B. Carrasco, S. Ayora and J. C. Alonso: *Bacillus subtilis* RecO nucleates RecA onto SsbA-coated single-stranded DNA. *J Biol Chem*, 283(36), 24837-47 (2008) doi:M802002200 [pii] 10.1074/jbc.M802002200
16. R. Torres, E. Serrano and J. C. Alonso: *Bacillus subtilis* RecA interacts with and loads RadA/Sms to unwind recombination intermediates during natural chromosomal transformation. *Nucleic Acids Res*, 47(17), 9198-9215 (2019) doi:10.1093/nar/gkz647
17. M. Gassel and J. C. Alonso: Expression of the *recE* gene during induction of the SOS response in Bacillus subtilis recombination-deficient strains. *Mol Microbiol*, 3(9), 1269-76 (1989) doi: 10.1111/j.1365-2958.1989.tb00277.x

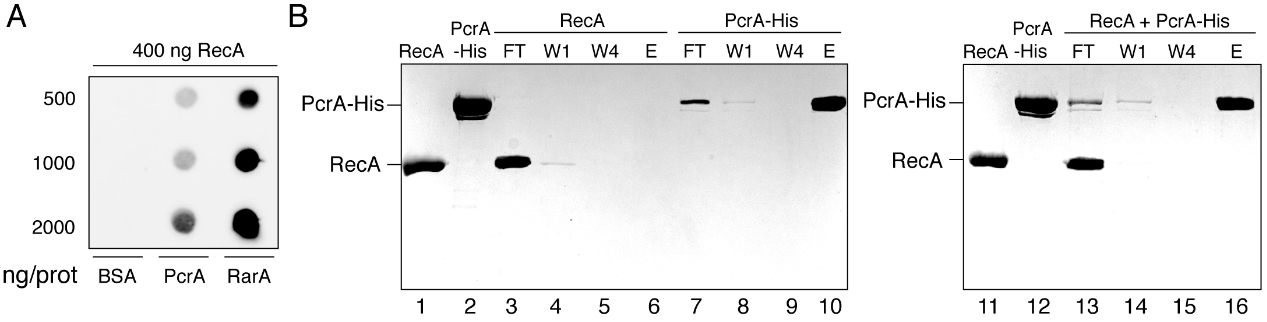

Figure S1. PcrA transiently interacts with RecA. (A) Immuno-dot blot of the PcrA interaction with RecA. Increasing PcrA, RarA (positive) and BSA (negative control) concentrations (500 to 2000 ng) were loaded onto a nitrocellulose membrane, RecA (400 ng) was incubated with the membrane, and subsequently washed. Polyclonal anti-RecA antibodies were used to highlight the protein-protein interactions, as described in Materials and Methods. Experiments were repeated three or more times with similar results, and a representative membrane is shown. (B) His-tagged PcrA (1 µg), RecA (1 µg) or His-tagged PcrA pre-incubated with RecA (5 min, 37 ºC) in buffer A (50 mM NaCl) were loaded onto a 50 µl Ni^{2+} matrix, and the FT was collected. The Ni^{2+} matrix was washed four times with 500 µl of buffer A containing 20 mM imidazole. Finally, bound His-tagged PcrA was eluted (E) with Buffer A containing 1 M NaCl and 0.4 M imidazole. Experiments were repeated three or more times with similar results, and a representative gel is shown.

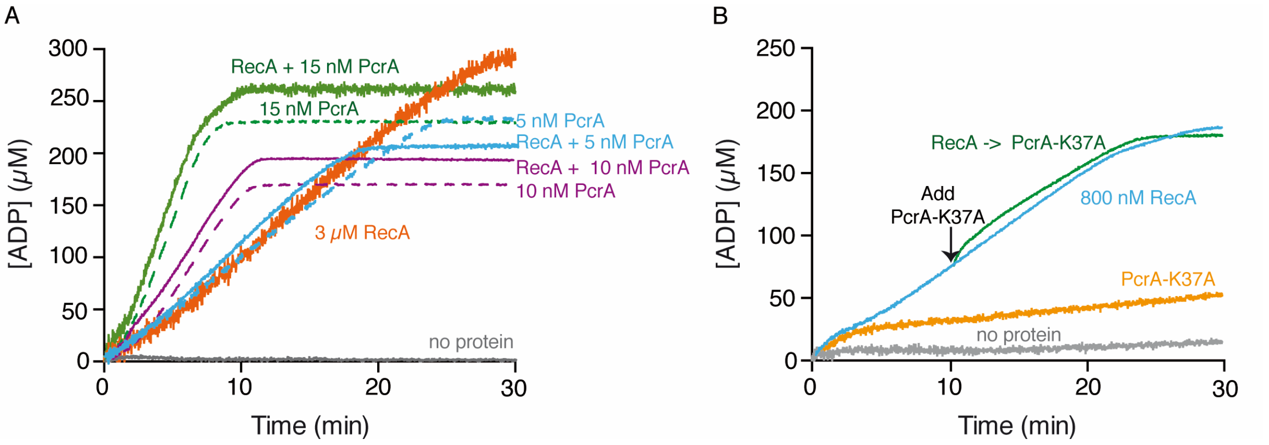

Figure S2. PcrA strips RecA·ATP filaments by loading onto ssDNA vacated by RecA·ADP. (A) PcrA (15 nM), RecA (3 μM) or both proteins was (were) incubated with cssDNA (10 μM in nts) in buffer A containing 5 mM ATP, and the ATPase activity was measured (30 min, 37 ºC). The broken lines denote the presence of only PcrA, at the indicated concentration. (B) PcrA K37A (15 nM) was incubated or not with cssDNA. The cssDNA was pre-incubated with RecA (800 nM) (10 min, 37 ºC) in buffer A containing 5 mM ATP, then PcrA K37A was added and the ATPase activity was measured (30 [or 20] min, 37 ºC). Buffer A contained the NADH enzyme mix. The gray line denotes the control reaction corresponding to the ATPase assay in the absence of any protein added (A-B). Representative graphs are shown here, and the determined K_{cat} is described in the text.

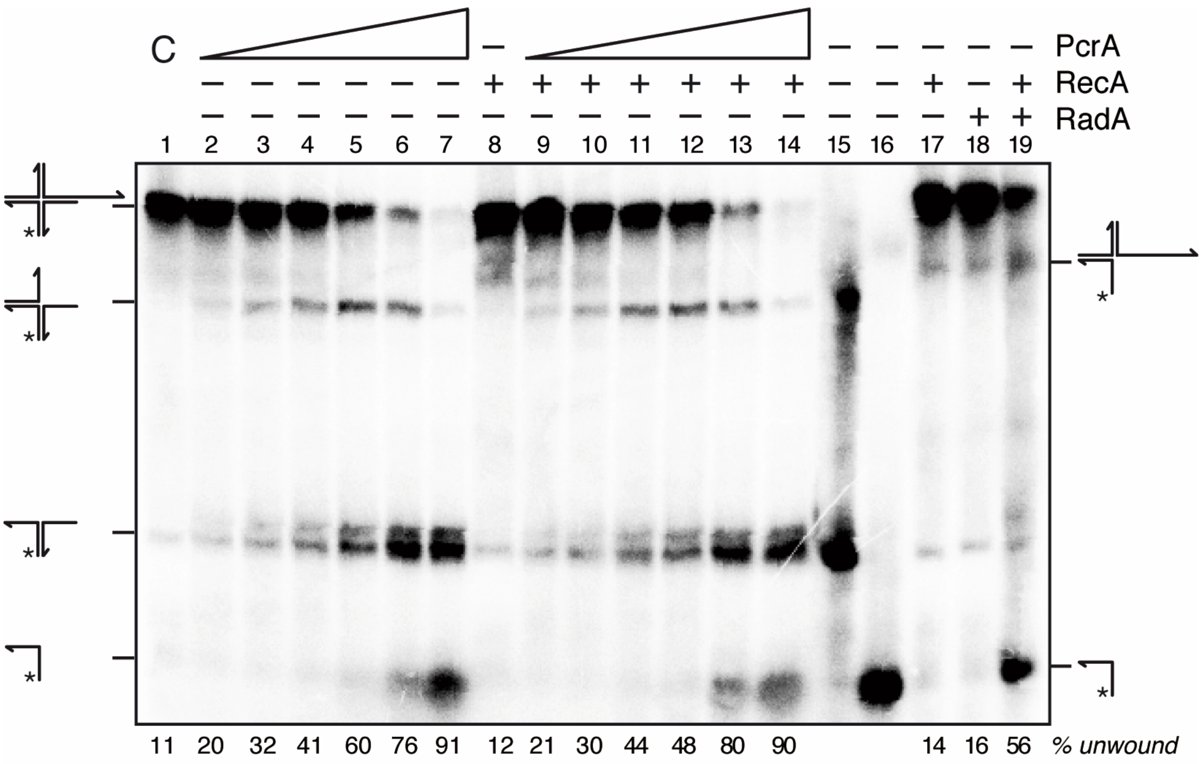

Figure S3. PcrA displaces RecA from HJ DNA with a longer nascent leading-strand. Cartoons illustrating substrate, intermediates and product upon incubation with PcrA or RadA/Sms are shown. The 3´-tail HJ DNA (0.5 nM in molecules) was incubated with increasing PcrA (0.35 to 12 nM), increasing PcrA and fixed RecA (400 nM) or fixed RecA and RadA/Sms (20 nM) concentrations; and the helicase activity measured. Reactions were done in buffer A containing 5 mM ATP (15 min, 30 ºC), and after deproteinization the substrate and products were separated by 6% PAGE and visualized by phosphor imaging. The mean quantification values of unwound DNA from >3 independent experiments are documented. Abbreviations: C, control 3´-tail HJ DNA substrate in the absence of any protein; - and +, absence and presence of the indicated protein; * denotes the labelled strand.

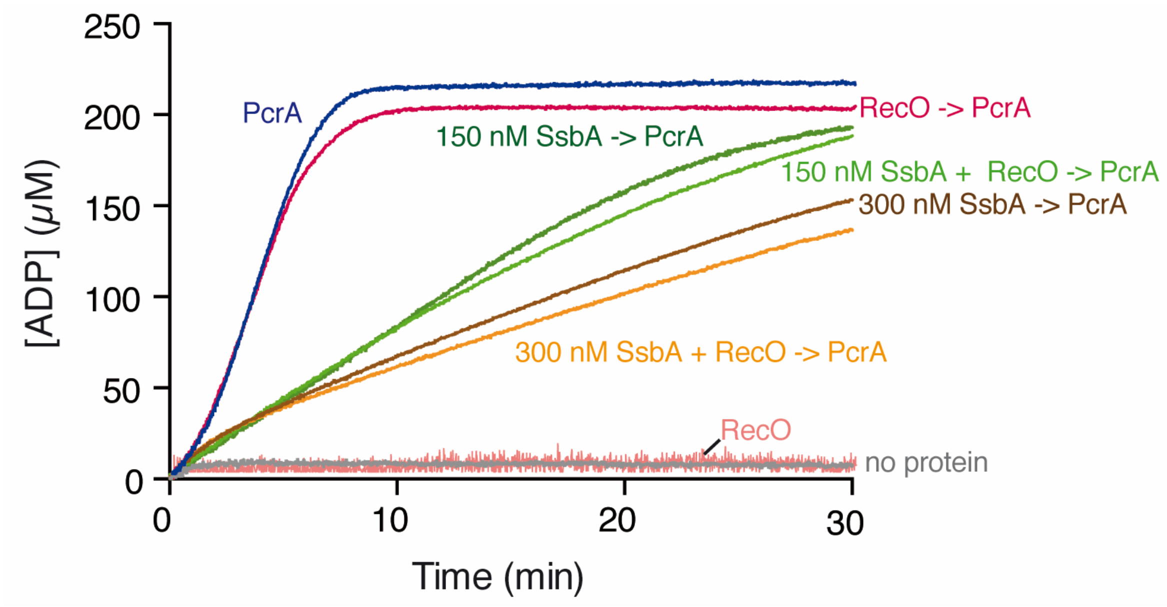

Figure S4. PcrA ATPase activity on csDNA in the presence of RecO. The cssDNA (10 μM in nts) was incubated with PcrA (15 nM), RecO (100 nM) or increasing SsbA concentrations (150 - 300 nM), or both fixed RecO and/or increasing SsbA concentrations was(were) pre-incubated with cssDNA (5 min, 37 ºC) in buffer A and then PcrA and 5 mM ATP and the NADH enzyme mix were added and the ATPase activity was measured (30 min, 37 ºC). The grey line denotes the control reaction corresponding to the ATPase assay in the absence of any protein added and the pink line in the presence of RecO. Representative graphs are shown here, and the determined K_{cat} is described in the text.

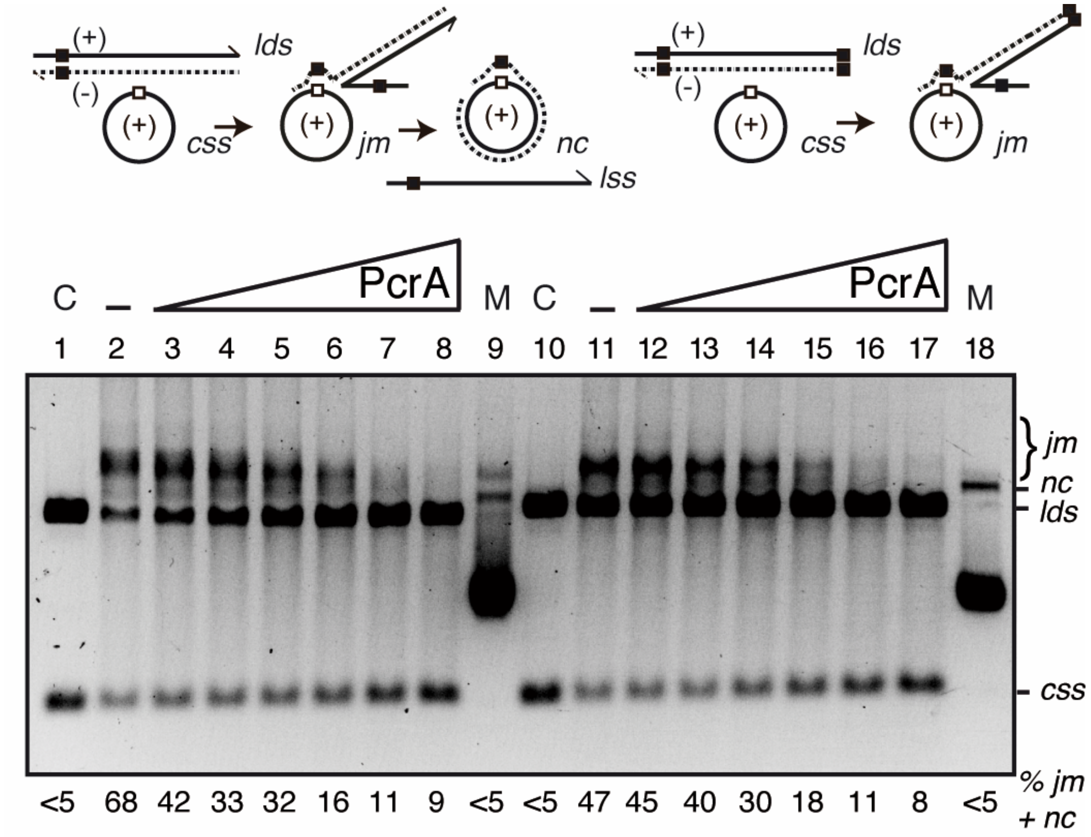

Figure S5. PcrA inhibits RecA-mediated DSE independently on the 3′→5′polarity. Cartoon of the reaction between *css*$_{hom}$ (empty square), *lds*$_{het}$ (black square) and the *lds*$_{het\text{-}ins}$ (black squares) substrates with internal heterologous regions towards the 3′ end (lanes 2-8) and the 3′ end and 5′-end (lanes 11-17). The *css*$_{hom}$ DNA (10 µM in nt) was preincubated with SsbA (300 nM) and RecO (100 nM) (5 min, 37 °C) in buffer A. Then, increasing PcrA (3 - 100 nM), and fixed RecA (800 nM), *lds*$_{het}$ (lanes 2 -8) or *lds*$_{het\text{-}ins}$ (lanes 11-17) and ATP (5 mM) concentrations were added, and the reaction was incubated (60 min, 37ºC). The percentage of *jm* intermediates and *prd* products are shown beneath the gel as the percentage of total substrate added. Lane 1, the *css* and the *lds*$_{het}$ or *lds*$_{het\text{-}ins}$ substrates (termed C) are indicated and in lanes 9 and 18 nicked circular 3276-bp pGEM $_{het}$ (lane 9) or 3353-bp pGEM3$_{het\text{-}ins}$ (lane 18) plasmid DNA was added as a mobility control of the *nc* product. The position of the bands and substrates are described in Figure 4. The results are the average value obtained from more than three independent experiments and a representative gel is shown here.
